# Pollinator restoration in Brazilian ecosystems relies on a small but phylogenetically-diverse set of plant families

**DOI:** 10.1038/s41598-019-53829-4

**Published:** 2019-11-22

**Authors:** Alistair John Campbell, Luísa Gigante Carvalheiro, Markus Gastauer, Mário Almeida-Neto, Tereza Cristina Giannini

**Affiliations:** 1 Instituto Tecnológico Vale, Belém, Brazil; 2 Embrapa Amazônia Oriental, Belém, Brazil; 3 Universidade Federal do Goias, Departamento de Ecologia, Goiânia, Brazil; 4 Universidade Federal do Pará, Belém, Brazil; 5 Centre for Ecology, Evolution and Environmental Changes, Universidade de Lisboa, Lisboa, Portugal

**Keywords:** Biodiversity, Conservation biology, Ecological networks, Restoration ecology

## Abstract

The alarming rate of global pollinator decline has made habitat restoration for pollinators a conservation priority. At the same time, empirical and theoretical studies on plant-pollinator networks have demonstrated that plant species are not equally important for pollinator community persistence and restoration. However, the scarcity of comprehensive datasets on plant-pollinator networks in tropical ecosystems constrains their practical value for pollinator restoration. As closely-related species often share traits that determine ecological interactions, phylogenetic relationships could inform restoration programs in data-scarce regions. Here, we use quantitative bee-plant networks from Brazilian ecosystems to test if priority plant species for different restoration criteria (bee species richness and visitation rates) can be identified using interaction networks; if phylogenetic relationships alone can guide plant species selection; and how restoration criteria influence restored network properties and function. We found plant species that maximised the benefits of habitat restoration for bees (i.e., generalists and those with distinct flower-visitor species) were clustered in a small number of phylogenetically-diverse plant families, and that prioritising the recovery of bee visitation rates improved both stability and function of restored plant-pollinator networks. Our approach can help guide restoration of pollinator communities, even where information on local ecosystems is limited.

## Introduction

Growing concern over the negative environmental impacts of human activities has put ecological restoration at the forefront of the fight to reverse biodiversity loss and to recover ecosystem functions and services^1^. Historically, restoration programs have focused on structural indicators, such as the recovery of plant communities relative to a reference ‘pristine’ state (e.g., pre-European settlement)^2^, with the assumption that fauna and ecosystem processes (e.g., pollination, seed dispersal, nutrient cycling) recover in tandem^3^. However, more recent evidence has demonstrated that selecting plant species to restore biotic interactions, rather than habitats, is critical to maintain ecosystem functions and health^4–7^.

Animal-mediated pollination is an essential ecological function in most terrestrial ecosystems^8^. Among pollinator taxa, bees are considered to be the most important group in both natural and agricultural ecosystems^9,10^. Unfortunately, many species are sensitive to anthropogenic disturbance, particularly the loss and fragmentation of natural habitats, and are globally in decline^10,11^. Outside of agricultural systems, for which there is considerable evidence on the benefits of habitat restoration for wild bees and crop pollination services (e.g. wildflower strips, hedgerows)^12–14^, bees are rarely considered as an explicit restoration target in natural lands^3,15^. Given that one of the key aims put forward by the International Union for Conservation of Nature’s (IUCN) Bonn Challenge (www.bonnchallenge.org) is to restore 350 million hectares of degraded land by 2030, a failure to target bees could lead to reproductive failure and even collapse of plant communities in restored habitats^15^.

To investigate the impacts of habitat restoration on bees, many studies adopt network-based approaches, as they provide quantitative and comprehensive information on how environmental changes spread through ecological communities and influence their stability and function^4,16^. Studies on plant-bee networks (and more broadly “plant-pollinator networks”) reveal that network generalists, species with many interaction partners, form the core of these networks, and are essential for their dynamic and structural stability^17,18^. These findings have important implications for ecological restoration, and several network-based measures of species-level generalisation, alongside modularity (i.e. ‘hubs’ and ‘connectors’), and ecological distinctiveness indices (i.e. functional complementarity), have been put forward to select plant species for bees^18–23^. In recent years, conservation scientists have also adopted machine-learning techniques (e.g. genetic algorithms) to inform plant species selection in regional bee restoration programs^24^. However, together with uncertainty over which approach provides the best outcome for bees and pollination, a major drawback of network-based restoration is the need for accurate and meaningful data on interspecific interactions^7^. At present, plant-pollinator networks in countries in tropical regions, such as Brazil, remain limited in number, and overrepresented by vertebrate pollinators (e.g. hummingbirds, bats)^25,26^. This data-deficiency is compounded by accelerating impacts of human activities in the tropics relative to other global regions^27,28^.

In data-scarce regions, restoration managers could use phylogenetic relationships among plant species as proxies of ecological function^29^. Phenotypic complementarity between interaction partners (e.g. long-tonged bees visit deep corolla flowers) has been found to be an important predictor of ecological network structure and function (e.g. pollination)^30–32^, although much of the existing data comes from temperate rather than tropical ecosystems. Because closely-related species tend to have similar functional traits, due to shared ancestry^33–35^, they often occupy similar positions in networks, both in terms of number of interaction partners and their partner species’ functional attributes^34,36^. This trait conservation within evolutionary lineages has great potential for use in bee restoration^37^. For example, if priority plant species (i.e. those that support high diversity/abundance of bees) occupy defined positions within the phylogeny, land managers could adapt findings from existing networks to habitats with slightly different species compositions^38^. Such an approach could facilitate the recovery of bee communities in degraded habitats at large spatial scales and across heterogeneous environments.

Here, we use bee-plant networks from across Brazil to test if priority plant species for different restoration criteria (bee species richness and bee visitation rates) can be identified using species-level metrics (for details on chosen network metrics, see Table 1). We compare bee community recovery (i.e. restoration success) under different metrics to outcomes using random plant species selection and machine-learning techniques (genetic algorithms), for quantitative networks sampled in two distinct physiognomies (forest or savannah-like biomes). We then use null models to test if phylogenetic relationships can identify priority plant species in regions for which no information on interaction patterns is available. To provide practical information for bee restoration managers, we additionally investigate whether these plant species are clustered within specific families. Finally, we evaluate if specific restoration criteria influence the structural properties of restored bee-plant networks, and the potential of restored bee species to pollinate wider plant communities using a proxy measure of pollination function (shared flower visitor index).

**Table 1:** Network metrics used to describe species-level generalisation and compositional differences in supported pollinator communities among plant species.

Species-level metric	Description	Citing references
i) Normalised Degree (ND)	The number of interaction partner species divided by total number of species in other trophic level	^17,19^
ii) Interaction Strength (ST)	Sum of partner species’ dependencies (proportion of visits to given plant species)	^39^
iii) Closeness Centrality (CC)	The proximity of individual nodes (species) to all other nodes within a network; species with high scores are important to many other species	^18,19^
iv) Betweenness Centrality (BC)	How individual nodes act as ‘connectors’, linking otherwise unconnected subsets (modules) of species	^18,19^
v) Functional Complementarity (FC)	The ecological distance between plant species’ pollinator assemblages	^22^

Cited references include studies that previously highlighted importance of metrics for restoration of mutualistic interaction networks.

## Results

### Selecting priority plant species for bee restoration using interaction networks

**Figure 1:**
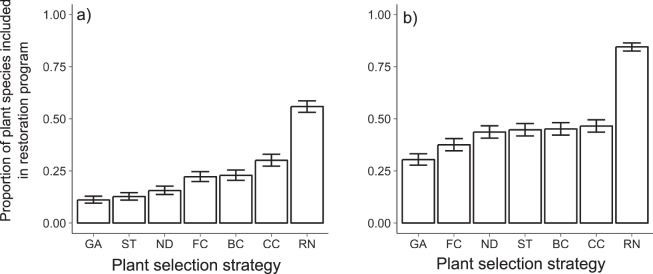
Proportion of plant species in networks included in restoration programs for two distinct criteria: a) maximise bee richness, and b) maximise bee visitation rates, under different plant selection strategies (BC – betweenness centrality, CC – closeness centrality, FC – functional complementarity, GA – genetic algorithms, ND – normalised degree, RN – random species selection, ST – strength). Error bars represent 95% confidence limits.

The number of plant species required to meet the bee species richness and bee visitation rate targets (80% of species/visits recovered) greatly depended on plant selection strategy (i.e. network metrics, genetic algorithms, or random selection), biome type (forested or savannah-like biomes), and network size (Table 2; for weighted model equations, see Table S1, Supplementary Information). As expected, network-based strategies outperformed random selection, requiring at least 50% fewer plant species (Fig. 1). However, only when bee species richness was targeted did a network metric (interaction strength) match the performance of machine-learning methods (genetic algorithms), requiring on average only 11% of plant species per network (Fig. 1a). For the recovery of bee visitation rates, no metric-based strategies performed better than the genetic algorithms, that required on average 20% fewer plant species than the best metric-based strategy – selection by functional complementarity (Fig. 1b). For both restoration criteria, a lower proportion of total plant species per network was required to meet restoration targets in savannah-like biomes compared with networks from forested biomes (Table S1). Results from networks excluding non-native honeybees (*Apis mellifera*) were qualitatively similar for both restoration criteria (Table S2).

**Table 2:** Model selection results on the effect of plant selection strategy (PS), biome type (BT) and network size (NS) on the proportion of plant species in bee-plant networks required to meet targets for two restoration criteria (maximise bee richness, maximise bee visitation rates).

Restoration criteria	PS	BT	NS	PS x NS	AICc	ΔAICc	Wgt
Bee richness							
Model 1	X	X	X		918.37	0.00	0.44
Model 2	X		X		918.77	0.41	0.36
Model 3	X	X			920.01	1.64	0.19
Relative Importance (*w*)	1.00	0.64	0.81				
Average Model	Y = Intercept + PS + BT + NS						
Bee visitation							
Model 1		X		X	930.62	0.00	0.34
Model 2				X	931.09	0.47	0.27
Model 3	X	X			931.43	0.81	0.23
Model 4	X	X	X		932.18	1.56	0.16
Importance (*w*)	1.00	0.73	0.77	0.62			
Average Model	Y = Intercept + PS + BT + NS + NS:PS						

Models were fitted using general linear mixed models assuming binomial distribution and, for each criteria, the table shows the most parsimonious models (∆AICc < 2). Model weight (Wgt) shows strength of evidence for each selected model and fixed effects included in selected models are indicated by cross (for parameter estimates, see Table S2). Relative Importance (*w*) of fixed effects and full equation of weighted (average) models are also presented.

### Phylogenetic relationships among priority plant species

**Figure 2:**
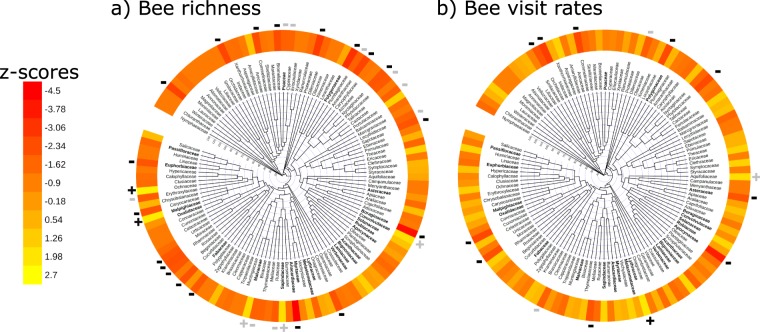
Phylogenetic tree of angiosperm families included in plant-bee networks and importance values for each plant family (i.e. z-score of the difference between observed and expected number of species selected) that could be selected as priorities for restoration programs to recover a) bee species richness, and b) bee species visitation rates. Plant families included significantly more or less often than expected by chance are indicated by ‘ + ’ and ‘ − ’ symbols; with significant (P < 0.05) and marginally-significant (P < 0.10) differences indicated in bold and light grey, respectively. The names of common plant families (>10 species occurrences in networks) are shown in bold.

Results from two measures of phylogenetic structure (Net Relatedness Index – NRI, and Nearest Taxon Index - NTI) revealed that, across the global species pool (Table 3), but not in individual networks (Table S3, Supplementary Information), plant species selected for restoration criteria were phylogenetically-clustered compared with random samples of equivalent size (null models). Results from full networks and those excluding honeybees showed that phylogenetic clustering as measured by the NTI was more often significant (3 out of 4 cases) than the NRI (1 out 4 cases) (Table 3). This implies that priority plant species occurred in several clusters throughout the phylogeny, rather than in closely-related families (Fig. 2; Fig. S1 – without honeybees), and that for individual networks, a small number of species per cluster would be sufficient for achieving restoration targets. Finally, phylogenetic turnover between biomes did not differ, indicating that compositional differences between forest and savannah-like biomes occurred mainly among lower taxonomic levels (i.e. genus/species) (Fig. S2).

**Table 3:** Level of phylogenetic clustering of plant species selected from global species pool (No. species) to meet restoration targets for two criteria: 1) maximise bee species richness, or 2) bee visitation rates.

Restoration criteria	No. species	(NRI)	*P*	(NTI)	*P*
Bee richness					
All bees	120	−1.833	0.034	−3.255	**0.002**
Without *A. mellifera*	121	−2.721	**0.001**	−1.021	0.174
Bee visitation					
All bees	340	−1.001	0.201	−2.228	**0.010**
Without *A. mellifera*	315	−1.087	0.158	−3.102	**0.001**

Phylogenetic structure assessed by negative Net Relatedness Index (NRI) and negative Nearest Taxon Index (NTI). In both cases, negative scores indicate observed sample is more phylogenetically clustered than expected values derived from weighted null models (*α* = 0.025). Results are presented from full networks and those excluding visits by honeybees (*A. mellifera*).

### Key plant families

Among common plant families (member species present in networks on ten or more occasions), Malpighiaceae was selected more often than expected by chance to recover bee richness (Fig. 2; full family list, Table S4, Supplementary Information). Nineteen families were selected less often than expected by chance, of which four were considered common in networks: Euphorbiaceae, Polygonaceae, Boraginaceae, and Anacardiaceae (Fig. 2; Table S4). As for bee visitation rates, one family, Lythraceae, was selected more often than expected by chance, and ten families, two being common (Apocynaceae and Asteraceae), were selected significantly less often than expected by chance (Fig. 2; Table S5). Results from networks excluding honeybees were qualitatively similar for the bee richness but differed when restoring bee visitation rates was the priority; only the Malpighiaceae (among common families) was selected significantly more often than expected by chance (Fig. S1, Tables S6 and S7).

### Evaluation of recovered network properties and pollination function

**Figure 3:**
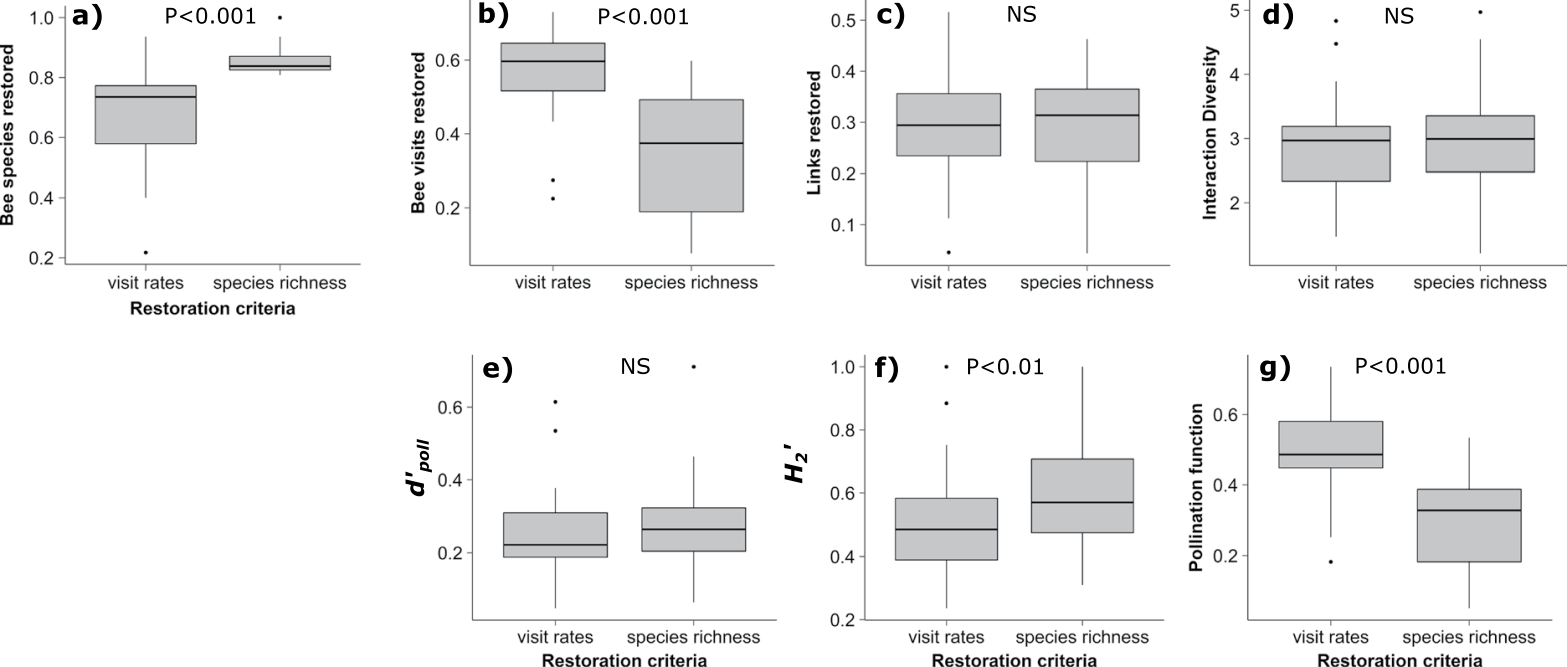
Effect of restoration criteria (bee visitation rates, bee species richness) on network structure and a proxy measure of pollination function. The following metrics were compared between networks of equivalent size (same number of plant species): (**a**) bee species richness, (**b**) bee visitation, (**c**) interaction richness (number of links), (**d**) interaction diversity, (**e**) bee species specialisation level (*d’*_poll_), (**f**) network level specialisation (*H*_2_′), and (**g**) pollination function (summed Müller index scores). Boxplots show median values and interquartile range. Significance values taken from results of likelihood ratio tests (see main text for details).

The use of different restoration criteria (bee species richness and bee visitation rates) resulted in restored networks differing in both the number of bee species and bee flower visits recovered (bee species - Likelihood Ratio Test (LRT) = 69.77, d.f. = 1, P < 0.001; bee visitation rates - LRT = 3402.80, d.f. = 1, P < 0.001), with restoration simulations that prioritised bee species richness outperforming those that prioritised visitation rates for the recovery of bee species richness, and vice versa for bee visitation rates (Fig. 3). The number of links recovered, interaction diversity (Shannon), and species-level (bees) specialisation indices in restored networks did not differ between criteria (Fig. 3). However, we found marginal and significant differences respectively, in network-level specialisation and the proxy measure of pollination function (potential contribution of restored habitats to wider plant communities via shared flower visitors), between networks constructed under different restoration criteria (*H*_2_′: LRT = 3.12, d.f. = 1, P = 0.077; pollination function: LRT = 52.35, d.f. = 1, P < 0.001). Network-level specialisation was reduced (more generalised), and the proxy measure of pollination function elevated in restoration simulations that prioritised the recovery of bee visitation rates versus simulations focused on bee species richness (Fig. 3).

## Discussion

Our results show that previous information on plant-bee interactions can be used to optimise restoration programs by the selection of plant species that maximise benefits to bee communities, and that priority species are clustered in a small number of phylogenetically-diverse plant families. We also found that prioritising the recovery of bee visitation rates over bee species richness, may be the most effective means of restoring ecosystem function in degraded Brazilian landscapes. Given the widespread impacts of human activities on tropical biodiversity, our results can help restoration practitioners to recover pollinator communities and ecosystem function in heterogeneous landscapes such as those found in Brazil, even where little is known beyond inventories of local plant communities.

Independent of which component of bee communities was targeted (species richness, bee visitation rates), the genetic algorithm tool developed by M’Gonigle *et al*.^24^ was consistently the most effective means of selecting plant species. Only in the case of bee species richness did a network metric (interaction strength) come close to matching results provided by the algorithms, reinforcing that generalist plant species, which are visited by disproportionately high numbers of bee species relative to other plant species, are essential for restoring bee diversity^17,39^. Despite the primacy of the algorithms, network indices may provide a better indication of the actual ecological drivers underpinning bee-plant network structure, and bee community recovery. For example, plant species selected by functional complementarity (FC)^22^, the best-performing metric for bee visitation rates, may reflect important functional differences between plant species (e.g. floral traits, resource type, phenology). As a consequence, this metric probably recovered discrete subsets of closely-interacting species (modules) faster than species-level generalisation metrics (e.g. degree, strength, centrality), that most likely selected plants with a high degree of overlap in their floral traits (e.g. short, open corollae) and visitor communities. However, plant species with high FC scores (i.e. distinct flower-visitor communities) could also include rare species that contributed little to overall bee visitation rates. In contrast, algorithms exclusively-selected plant species based on sum totals of visits recovered, a factor likely to be strongly influenced by an individual plant species’ relative abundance in sampled habitats^40^. This highlights the importance of standardising species’ abundances (e.g. visits/flowers) to disentangle the relative importance of functional traits and local abundance in structuring plant-pollinator networks, something that many existing plant-pollinator networks (but with some important exceptions^4,41,42^), do not account for.

An important caveat to our results is that aggregated topological models (>1 yr of repeat surveys) used to simulate the bee community recovery did not account for inherent temporal dynamics of plant-pollinator networks (i.e. temporal overlap in bloom/insect activity periods), nor the plastic behavioural responses of bees to the gain or loss of plant species (i.e. ‘rewiring’)^39^. Thus, it is unclear to what extent these aggregate networks can be used to predict flower-visitor communities in restored (i.e. partial) habitats. However, our aim was to identify plant species that provide the greatest benefit to bee communities (i.e. maximum number of species/visits), including potential rewiring species (e.g. bee species A only visits plant species B in the absence of plant species C), which can be largely determined using aggregated networks.

Across networks, phylogenetic clustering of priority species was strongest under the nearest taxon index (NTI), indicating that priority species were found in several clusters (i.e. families), rather than one large cluster of closely-related taxa, which would cause the net relatedness index (NRI) to indicate stronger clustering as well^43^. This demonstrates firstly, that phylogenetic relationships can be used to detect priority plant species for bee restoration, and secondly, by increasing phylogenetic diversity among selected species, we can likely reduce the number of plant species required (and hence costs) in restoration programs. This was supported by the lack of phylogenetic clustering among priority species in individual networks, which given that functional traits are expected to be conserved among closely-related species^34,35^, would lead to reduced levels of functional complementarity among selected plant species, a key driver of bee diversity in natural and agricultural habitats^44^. Furthermore, elevated competition among closely-related species may preclude co-occurrence at individual sites^45^, although this will depend on the relative importance of abiotic (i.e., environmental filtering) and biotic factors (e.g., interspecific interactions) in community assembly processes^35^. Finally, although biome type was an important factor in determining the relative number of plant species required to meet our restoration targets (higher in forested biomes), we found no evidence of significant phylogenetic turnover between biomes. Therefore, whilst species composition varied between networks in different biomes, overall phylogenetic composition and structure of plant communities did not, allowing our recommendations to be applicable to distinct Brazilian biomes. Nonetheless, many of the evaluated networks were from south-eastern Brazil, and further research is required in underrepresented regions, particularly Amazonia, which was represented by a single network (24 in total), to ensure findings are relevant for the restoration of Brazilian bees.

Considering only native bee taxa, assessments at family level revealed that members of the Malpighiaceae family were clear priorities for bee restoration in studied regions. Malpighiaceae species are well known for their importance to oil-collecting solitary bees^46^, that together represented 15% of bee species in networks. Collected oil is used as both adult and larval food, and in nest construction^46^. As well as their importance for oil-collecting taxa, several studies examining pollen stores of native eusocial bees have highlighted the importance of Malpighiaceae pollen, among other families, as larval food^47–49^. Thus, not only do Malpighiaceae flowers form highly specialised interactions with oil-collecting bees, they provide protein-rich pollen for generalist eusocial taxa, underlining their importance for Brazilian bee restoration.

Alongside the Malpighiaceae, several other families were selected more often than expected by chance (e.g. Convolvulaceae, Sapindaceae), and likely to be important priorities for bee restoration in Brazilian ecosystems, although they fell above the significance threshold (*α* = 0.05). Likewise, families which were selected less often (e.g. Anacardiaceae, Asteraceae), whilst still providing resources for flower-visiting bees, are considered low priorities for bee restoration programs. However, reasons why these families promote (or hinder) the recovery of bee communities, and the extent to which these differences are determined by specific functional traits shared among family members (e.g. elaiophores in Neotropical Malpighiaceae), or multiple traits working in chorus, and the relative importance of different traits across geographic regions, remains unresolved. Thus, future studies should aim to incorporate trait-based approaches to identify functional drivers of patterns observed here and test the validity of ‘trait-matching’ hypotheses in diverse tropical plant-pollinator networks. Despite these drawbacks, our results do provide an initial guide (i.e. key plant families) for Brazilian restoration managers, although, given the limited number of families positively or negative selected, further work is required to translate our findings into actual restoration practices. Eventually, this approach could also be adapted to agricultural settings with the aim of improving crop pollination services through the establishment of ‘bee-friendly’ habitats in croplands (e.g., hedgerows, wildflower plantings)^12,50^.

Overall bee community recovery and restoration of pollination function were dependent on the specific criteria being targeted in simulations (i.e., bee species richness or visitation rates). Simulations that prioritised the recovery of bee visitation rates over species richness tended to produce more generalised networks and had higher values for a proxy measure of pollination function (i.e., export of shared flower-visitor species to adjacent habitats). High levels of generalisation imply greater functional redundancy among interacting species, a positive driver of stability and function in mutualistic networks, and a key priority for ecological restoration^4^. However, given that we defined restoration success as the minimum number of plant species required to meet our *a priori* restoration criteria (80% of bee species/visits restored), further work is required to make sure that levels of functional redundancy among selected plant species are sufficient to ensure that restored bee communities and pollination services are resilient to future disturbances^4^.

Improved pollination function in simulations that targeted visitation rates may have been because plant species predominantly targeted hyper-abundant ‘supergeneralists’ (i.e., species that interact with disproportionately high numbers of plant species), such as the eusocial bee species *A. mellifera* and *Trigona spinipes*, that together represented 46.3% of total visits. Previous research has highlighted the importance of generalist species for network stability and function^17^, and more specifically, the contributions of the above mentioned bee species in Brazilian ecosystems^51^. Assuming recovered bee populations move between restored and adjacent habitats, restoration programs benefiting supergeneralists probably have the greatest potential to quickly recover ecosystem functions across large spatial scales. On the other hand, promoting supergeneralists over the recovery of bee species diversity may exacerbate growing problems of biotic homogenisation in tropical ecosystems^52^. Furthermore, caution is required in assuming that the Müller (‘shared pollinators’) index is a useful proxy of pollination function in wider plant communities. Firstly, it remains unclear the degree to which bees move between restored and adjacent habitats, a factor likely to be greatly influenced by bee foraging range, behaviour, nesting habit, and surrounding landscape structure^1^. Secondly, our models do not include information on pollinator effectiveness, or the dependence of plant species on biotic pollination^39^, and evidence from pollen deposition networks suggests that plant-pollinator interactions may be more specialised than they first appear in flower-visitor networks^53^. Therefore, just because restored plant species sustain bee species that also visit many other plant species does not necessarily imply that those insects are effective pollinators of all visited plant species. And finally, because bee-plant networks ignore the contributions of other insect flower visitors (e.g. flies, beetles, butterflies/moths), of which many are effective pollinators in tropical plant communities. Thus, enhancing native pollinator species diversity, as well as improving the overall conservation value (*A. mellifera* is an invasive species in Brazil), is likely to be an essential component in the recovery of pollination function in degraded Brazilian landscapes.

In this study we demonstrate that priority plant species for the restoration of Brazilian bee communities can be selected based on prior information on their interaction patterns. Not only are they largely generalist species that form many interactions with flower-visiting bees but are also non-randomly distributed across the angiosperm phylogenetic tree. This information, along with the identity of key plant families, can provide restoration practitioners with the means to select priority plant species for bee community recovery in degraded lands, even where ecological information on the target ecosystem is limited. Further studies evaluating restoration success in areas where these selection practices are applied *in loco* will be important to validate our model-based predictions.

## Material and Methods

### Bee-plant interaction networks

We used 24 quantitative bee-plant networks from Brazil constructed using standardised sampling methods^54^. All networks were found in published articles or publicly-available theses/dissertations (see Data Availability statement), and representative of communities in several biomes with distinct physiognomies, including Atlantic rainforest, *Restinga* coastal forest, and savannah-like habitats (*Cerrado* and *Caatinga*) (Table S8; Fig. S3 in Supporting Information). Networks from each sample site were considered independent as all sample sites were at least 2 km apart (except one – but conducted in different habitat physiognomies, see Table S8).

### Selecting priority plant species using interaction network metrics

Our aim was to find optimal combinations of plant species to maximise two components of bee communities: 1) bee species richness, and 2) visitation rates, respectively (henceforth ‘restoration targets’). We chose these targets as they are basic aims of most pollinator conservation actions, and are positively related to the stability and magnitude of pollination services^10,55^.

We used four species-level metrics of generalisation and one index of ecological distinctiveness in pollinator assemblages (functional complementarity) to inform plant selection in simulations of bee community recovery (for further details on metrics, see Table 1). All metrics unless otherwise stated were calculated using the *bipartite* package in *R* (ver. 2.08, Dormann *et al*. 2009). Topological models were used to simulate impacts of restored plant species on bee communities in cleared or abandoned habitats (‘from scratch’). During simulations, plant species were added in multiples of two using the functions ‘specaccum’ in *R* package *vegan* (v. 2.52)^56,57^ “and ‘cumsum’ (*R* base function)”, for bee richness and bee visitation rate models, respectively, until restoration targets were achieved. We chose 80% of bee species/visits from the original (‘pristine’) community as our restoration targets as pollination functions are largely provided by abundant generalist species^58^, that would likely be supported at such thresholds. Furthermore, additional testing of different thresholds (10 to 90%) revealed that only at such high thresholds do restored networks show robustness to random extinction of higher-level species comparative to that of full networks, and which provide bee flower visitors to the majority of plant species in networks (Fig. S4, Supplementary Information). Strategies that required the fewest number of plant species to meet our targets were considered the most effective strategies for bee restoration.

Results from network-based strategies were compared to mean values under random selection (1000 simulations for each multiple of two plant species), and those determined by a freely-available ‘genetic algorithm’ tool designed to select priority species from networks based on *a priori* restoration criteria (i.e., maximise bee richness)^24^. Results from random selection and genetic algorithms were respectively, considered sub-optimal and near-optimal solutions for each restoration target, and therefore means to evaluate bee community recovery under different network-based strategies.

### Statistical analyses

Recovery of bee communities under different plant selection strategies was evaluated using generalised linear mixed-effects models (GLMMs) with binomial errors using ‘glmer’ function in *lme4* package^59^. Our response variables were the number of ‘successes’ (plant species selected) and ‘failures’ (plant species not selected) in each network required to meet targets for bee richness and visitation rates (i.e., 80% of species/visits recovered from the original network). Thus, for each network response values closer to 0 require fewer plant species than those with values closer to 1. Fixed effects included plant selection strategy (i.e. network metrics, genetic algorithms, and random species selection), network size (number of unique pairwise interactions), biome type (‘savannah’ or ‘forest’ biomes), and all two-way interactions between predictor variables. Biome type was included in models because ordination analyses of plant communities (Non-Metric Multi-Dimensional Scaling and Analysis of Similarity) revealed that networks in forest biomes (Atlantic rainforest, Restinga) differed in their species composition from those in savannah-like biomes (Cerrado and Caatinga) (Fig. S5, Supplementary Information). Network was included as a random effect to account for non-independence of data from simulation models.

All candidate models were checked for collinearity using the function ‘max.r’ (https://github.com/rojaff/dredge_mc). Best-fitting models were selected from candidate model list using ‘dredge’ function in *R* package *MuMIn*^60^, where all models < 2 ΔAICc (corrected for small sample sizes) from top model were considered statistically equivalent^61^. Model residuals were visually checked for homogeneity of variance. Importance of fixed effects in explaining variation in response variables was assessed using relative importance values (sum of Akaike model weights across all models in which fixed effect occurs) and regression coefficients derived from average (weighted) models (<2 ΔAICc). All previous steps and those presented in sections below were also performed on networks excluding flower visits and plant species exclusively-visited by non-native honeybees (*A. mellifera*) (present in all but one of 24 networks).

### Phylogenetic relationships among priority plant species

To investigate the influence of phylogenetic relationships among priority plant species, we used different measures of relatedness for a given sample of species/individuals relative to the overall population mean, known as phylogenetic structure^43,45^. We tested phylogenetic structure of plant species combinations selected by the optimal plant selection strategy (fewest plant species required) for each of our two restoration targets. Phylogenetic structure analyses were performed on individual networks and all networks together (global species pool) to determine the scale at which phylogenetic relationships can inform pollinator restoration programs (for details on phylogenetic tree construction see Appendix I, Supplementary Information).

### Evaluating phylogenetic structure

To test whether priority plant species were randomly or non-randomly distributed across plant phylogenies, we used null models to generate 10 thousand random subsets of plant species of equivalent size to the optimum selection in individual networks and across all networks. Phylogenetic distances of observed (selected species) and null plant communities were used to compute: a) the negative Net Relatedness Index (NRI), calculated from the mean phylogenetic distance between all species pairs in a sample, and b) the negative Nearest Taxon Index (NTI), considering only the phylogenetic distance to the nearest species^43^. Results from NRI and NTI may or may not be correlated, depending on whether species clusters are ‘clumped’ together in closely-related families (correlation likely), or more widely-distributed across the phylogenetic tree (correlation unlikely)^43^.

Null models were constructed using unweighted (all species have equal probability of selection) and weighted procedures. Weighted models were constrained by plant ‘abundance’ (individual networks - number of bee visits; global species pool – sum number of species occurrences) using the *picante* package in *R*^62^. Results from both null model approaches were qualitatively similar, and so only those from weighted models are presented. One-sample z-tests were used to test differences between observed and expected levels of phylogenetic structure. As plant species composition differed between forest and savannah biomes, we also tested degree of phylogenetic turnover between biomes by comparing mean phylogenetic distances between network pairs using the R package ‘*picante*’ and function *comdist*^62^. We then used ANOSIM to test if mean phylogenetic distances within biome groups were smaller than between groups.

### Identification of key plant families

To determine key plant families for bee restoration, we first estimated the expected number of species to be selected as restoration priorities from each family based on their relative frequency in individual networks. For example, if 25% of plant species in a network belong to Family A, and 12 plants are selected for restoration, we expect that Family A will be represented by three species. We repeated this process for all 121 families and all networks and then used one-sample z-tests to compare expected (null) values, i.e. in the absence of ecological factors, with observed values (actual number of species selected). Where confidence intervals did not overlap 0 indicated a plant family that was selected more/less often than expected by chance. We included the additional caveat that families must be represented at least 10 times in networks to be considered relevant for restoration.

### Evaluation of recovered network properties and pollination function

We compared the effectiveness of bee community recovery (i.e., pollination of the wider plant community) and the stability of networks constructed under different restoration targets. As networks size differed between restoration targets (see results), we standardised the number of plants in individual networks to that required to meet the bee richness target (least number of plant species). For all recovered networks, we compared the following metrics: total bee richness, total visitation, interaction richness (number of links), interaction diversity (exponent of Shannon entropy *H*_2_), specialisation level of bee species (*d’*_*poll*_), and network-level specialisation (*H*_2_′). Total number of species, visits and interactions in restored networks were converted into proportions based on values from full networks (e.g. 10 of 20 bee species restored = 0.5). Differences between networks recovered under different restoration criteria (bee species richness or visitation rates) were assessed using linear mixed effects models (*H*_2_) and binomial GLMMs, with network included as a random effect. Significance of fixed effects were assessed using likelihood ratio tests.

To calculate a proxy measure of the potential contribution of restored bee communities to pollination function (including pollination services) in both restored habitats and wider plant communities, we estimated the average contribution of each restored plant species to the pollination of other plant species via shared flower visitors. To do that, we use an index developed by Müller *et al*.^63^ to assess apparent competition between resource species via shared consumers. Specifically, the index quantifies how much an individual plant species (‘acting species’) contributes to the diet of the consumers (pollinators) of another plant species (‘target species’)^64^. Values close to 1 signify that the acting species contributes greatly to the diet of the target species’ pollinators (sum of dependencies), and scores given to individual plant species represent the sum value across all species pairs in which that plant is the acting species. Müller index (MI) scores were calculated using the function ‘PAC’ in the R package *bipartite*. We summed scores for all plant species selected for different restoration targets and divided these by scores from full networks to provide a proxy measure of how much ‘pollination function’ was recovered under different scenarios. Differences in MI scores were assessed using a binomial GLMM with network included as a random effect. Significance of fixed effects were assessed using likelihood ratio tests.

## Supplementary Information

Supplementary Information

## Data Availability

All datasets used in this publication are freely available at the following website: *Scientific information system about neotropical bees*. http://abelha.cria.org.br/index
