# Supplementary material for: Pollinator restoration in Brazilian ecosystems relies on a small but phylogenetically-diverse set of plant families
Source: Sci Rep. 2019 Nov 22;9:17383. doi: 10.1038/s41598-019-53829-4 (PMC6874649; doi:10.1038/s41598-019-53829-4)
Supplement: Supplementary file 1 — Supplementary Information [file 41598_2019_53829_MOESM1_ESM.pdf]

Supplementary Information:

Pollinator restoration in Brazilian ecosystems relies on a small but phylogenetically-diverse set of plant families

Alistair John Campbell, Luísa Gigante Carvalheiro, Markus Gastauer, Mário Almeida-Neto, and Tereza Cristina Giannini

Table S1. Regression equations from average models (ΔAICc <2) of the number of plant species required to meet bee richness and bee visitation rate restoration targets (binomial GLMMs). Model parameters include: PS = Plant Selection strategy (BC - Betweenness Centrality, CC - Closeness Centrality, FC - Functional Complementarity, ND - Normalised Degree, RN - Random species selection, ST - Strength); BT = Biome Type (Forest or Savannah); and NS = Network Size. All parameter estimates are transformed (logit) and reference levels (Intercept) show parameter estimates from genetic algorithms (GA).

Restoration target			
i) Bee species richness			
(ΔAICc<2 = 3 models)	Full equation	Y = Intercept + PS + BT + NS	
	PS	Forest biomes	Savannah biomes
	Intercept	Y = -1.920 - 0.340*NS	Y = -2.481 - 0.340*NS
	BC	Y = -1.054 - 0.340*NS	Y = -1.615 - 0.340*NS
	CC	Y = -0.681 - 0.340*NS	Y = -1.242 - 0.340*NS
	FC	Y = -1.092 - 0.340*NS	Y = -1.653 - 0.340*NS
	ND	Y = -1.526 - 0.340*NS	Y = -2.088 - 0.340*NS
	RN	Y = 0.400 - 0.340*NS	Y = -0.161 - 0.340*NS
	ST	Y = -1.767 - 0.340*NS	Y = -2.328 - 0.340*NS
ii) Bee visitation rates			
(ΔAICc<2 = 4 models)	Full equation	Y = Intercept + PS + BT + NS + NS:PS	

PS	Forest biomes	Savannah biomes
Intercept	Y = -0.697 - 0.079*NS	Y = -1.037 - 0.079*NS
BC	Y = -0.094 - 0.18*NS	Y = -0.434 - 0.18*NS
CC	Y = -0.041 - 0.192*NS	Y = -0.381 - 0.192*NS
FC	Y = -0.371 - 0.053*NS	Y = -0.711 - 0.053*NS
ND	Y = -0.159 - 0.197*NS	Y = -0.499 - 0.197*NS
RN	Y = 1.798 - 0.188*NS	Y = 1.458 - 0.188*NS
ST	Y = -0.091 - 0.107*NS	Y = -0.431 - 0.107*NS

Table S2. Model selection results on the effect of plant selection strategy (PS), biome type (BT) and network size (NS) on the proportion of plant species in bee-plant networks required to meet targets for two restoration criteria (maximise bee richness, maximise bee visitation rates) where *Apis mellifera* and plants exclusively visited by this species have been excluded. Models were fitted using general linear mixed models assuming binomial distribution and, for each criteria, the table shows the most parsimonious models (ΔAICc <2). Model weight (Wgt) shows strength of evidence for each selected model and fixed effects included in selected models are indicated by cross. Relative Importance (w) of fixed effects and full equation of weighted (average) models are also presented.

**Restoration criteria**	**PS**	**BT**	**NS**	**PS x NS**	**AICc**	**ΔAICc**	**Wgt**
Bee richness							
Model 1	X	X		X	932.29	0.00	0.62
Model 2	X			X	933.26	0.97	0.38
w	1.00	0.62	1.00	1.00			
Average model	Y = Intercept + PS + BT + NS + NS:PS						
Bee visitation							
Model 1	X	X			886.88	0.00	0.31
Model 2	X		X		887.27	0.39	0.26
Model 3	X	X	X		887.56	0.69	0.22
Model 4	X				887.66	0.78	0.21
w	1.00	0.53	0.48				
Average model	Y = Intercept + PS + BT + NS						

Table S3. Phylogenetic clustering in individual networks weighted by plant abundance (sum visits). NRI = Net Relatedness Index, NTI = Nearest Taxon Index. Significant values highlighted in bold (p-values are two-tailed – must be < 0.025 or > 0.975 to be significant). Plants = number of plant species selected.

	Bee species richness					Bee visitation				
**Network**	**Plants**	**NRI**	***P***	**NTI**	***P***	**Plants**	**NRI**	***P***	**NTI**	***P***
net01	4	-0.525	0.210	-0.339	0.254	10	0.439	0.592	0.254	0.558
net02	6	0.716	0.746	0.640	0.682	14	-0.148	0.403	1.218	0.887
net03	4	0.471	0.516	-0.137	0.391	8	0.883	0.858	0.722	0.724
net04	10	-1.033	0.139	-0.719	0.235	18	0.112	0.598	-0.335	0.372
net05	6	-1.024	0.139	-1.016	0.155	12	-0.112	0.421	-0.285	0.391
net06	4	1.043	0.911	1.194	0.857	12	0.466	0.630	-0.669	0.244
net07	4	-0.449	0.213	-1.310	0.157	6	-0.011	0.357	-1.180	0.104
net08	4	0.135	0.485	0.465	0.604	10	0.449	0.646	0.641	0.738
net09	12	-1.723	0.045	-2.741	**0.004**	16	0.098	0.518	0.877	0.805
net10	8	-0.588	0.263	-0.074	0.457	16	0.326	0.635	0.163	0.552
net11	10	0.282	0.553	-0.501	0.301	28	-0.358	0.338	-1.563	0.054
net12	2	-0.669	0.117	-0.669	0.117	27	-0.352	0.357	0.538	0.703
net13	4	0.486	0.653	0.508	0.724	12	0.845	0.808	0.641	0.739
net14	6	-1.794	0.053	-1.396	0.095	12	1.096	0.891	1.610	0.969
net15	12	-1.147	0.118	-0.166	0.418	28	-0.641	0.251	-0.163	0.446
net16	4	-0.731	0.198	-0.680	0.228	8	-0.846	0.173	-0.686	0.240
net17	1	0.618	0.541	0.618	0.541	6	-0.188	0.411	-0.352	0.342
net18	4	0.338	0.627	-0.686	0.252	14	-0.284	0.347	-0.490	0.303
net19	4	0.614	0.621	0.344	0.439	12	0.341	0.601	0.162	0.556
net20	4	-0.238	0.332	-0.735	0.220	12	-0.284	0.409	-0.222	0.418
net21	6	-1.729	0.051	-0.835	0.199	12	-0.218	0.349	0.229	0.563
net22	8	0.070	0.501	-0.225	0.391	20	-0.978	0.163	-0.035	0.475
net23	4	0.222	0.497	0.736	0.689	20	-1.183	0.124	-1.772	0.039
net24	14	-0.057	0.457	-0.340	0.377	81	-0.689	0.247	-1.257	0.103

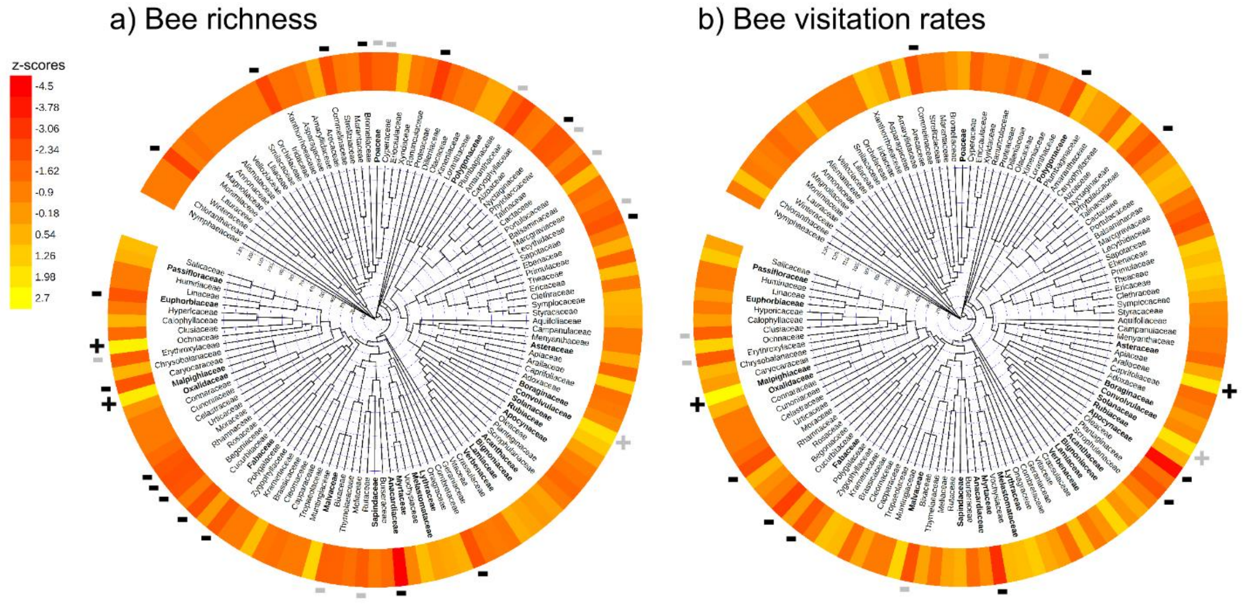

Figure S1. Phylogenetic tree of angiosperm families included in plant-bee networks and differences in each family's observed and expected (z-scores) selection as priorities for restoration programs to recover a) bee species richness, and b) bee visitation rates (visits of non-native *Apis mellifera* excluded from networks). Plant families included in priority species lists in individual networks significantly more or less often than expected by chance are indicated by '+' and '-' symbols; with significant ($P < 0.05$) and marginally-significant ($P < 0.10$) differences indicated in bold and light grey, respectively. The names of common plant families (> 10 species occurrences in networks) are shown in bold.

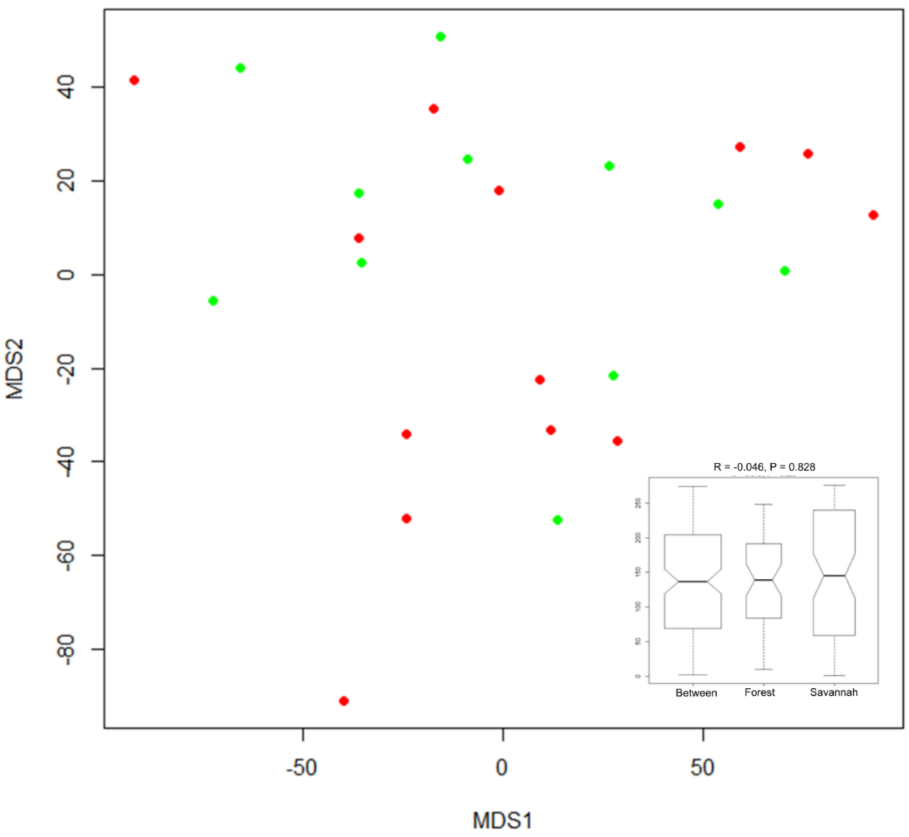

Figure S2. Principal Coordinates Analysis (PCoA) of mean phylogenetic distances between plant communities belonging to two distinct biome types: 'forest' (red dots) and 'savannah-like' (green dots). Ordination analyses were weighted by plant abundance (insect visitation rates used as proxy). Inset panel shows results from analysis of similarity (ANOSIM).

Table S4. Results from *z*-score analyses of expected and observed selection of 121 families in bee-plant networks for recovery of bee richness. Common families (at least 10 species occurrences) are listed in bold. Families with confidence intervals that do not overlap zero selected significantly more/less than expected by chance.

No.	Family	Occ.	*z*-score	*P*	Ave. diff.	-95% CI	+95% CI
1	**Acanthaceae**	14	0.09	0.926	0.00	-0.06	0.07
2	Adoxaceae	1	-1.00	0.317	0.00	0.00	0.00
3	Aizoaceae	1	-1.00	0.317	0.00	0.00	0.00
4	Alismataceae	2	-1.00	0.317	-0.01	-0.04	0.01
5	Amaranthaceae	8	-2.51	0.012	-0.03	-0.05	-0.01
6	Amaryllidaceae	1	-1.00	0.317	0.00	0.00	0.00
7	**Anacardiaceae**	26	-4.48	0.000	-0.11	-0.15	-0.06
8	Annonaceae	1	-1.00	0.317	0.00	-0.01	0.00
9	Apiaceae	3	-1.44	0.149	-0.01	-0.03	0.00
10	**Apocynaceae**	22	0.10	0.919	0.00	-0.08	0.09
11	Aquifoliaceae	5	0.22	0.824	0.01	-0.07	0.09
12	Araliaceae	7	0.84	0.403	0.04	-0.06	0.15
13	Arecaceae	9	1.00	0.319	0.05	-0.05	0.15
14	Asparagaceae	7	-1.40	0.162	-0.02	-0.04	0.01
15	**Asteraceae**	182	-0.22	0.823	-0.03	-0.29	0.23
16	Balsaminaceae	3	-1.39	0.164	0.00	-0.01	0.00
17	Begoniaceae	6	-1.00	0.317	-0.02	-0.05	0.02
18	**Bignoniaceae**	41	-0.17	0.863	-0.01	-0.17	0.15
19	Bixaceae	3	-1.76	0.079	-0.01	-0.02	0.00
20	**Boraginaceae**	16	-4.06	0.000	-0.06	-0.09	-0.03
21	Brassicaceae	3	0.87	0.385	0.03	-0.04	0.09
22	Bromeliaceae	5	-2.30	0.021	-0.02	-0.04	0.00
23	Burseraceae	2	-1.43	0.154	-0.01	-0.02	0.00
24	Cactaceae	7	-1.75	0.081	-0.03	-0.07	0.00
25	Calophyllaceae	3	-1.35	0.178	-0.03	-0.07	0.01
26	Campanulaceae	1	-1.00	0.317	-0.01	-0.02	0.01
27	Capparaceae	4	-1.35	0.178	-0.02	-0.04	0.01
28	Caprifoliaceae	2	-1.21	0.225	0.00	-0.01	0.00
29	Caryocaraceae	4	-2.07	0.039	-0.02	-0.04	0.00
30	Caryophyllaceae	2	-1.14	0.254	-0.01	-0.01	0.00
31	Celastraceae	1	-1.00	0.317	0.00	-0.01	0.00
32	Chloranthaceae	1	-1.00	0.317	0.00	-0.01	0.00
33	Chrysobalanaceae	5	0.64	0.524	0.02	-0.05	0.10
34	Cleomaceae	2	-1.14	0.255	-0.01	-0.01	0.00
35	Clethraceae	1	-1.00	0.317	0.00	-0.01	0.00
36	Clusiaceae	3	-1.43	0.153	-0.01	-0.02	0.00
37	Combretaceae	4	-2.13	0.033	-0.02	-0.03	0.00
38	Commelinaceae	6	-2.34	0.019	-0.02	-0.04	0.00
39	Connaraceae	1	-1.00	0.317	0.00	-0.01	0.00
40	**Convolvulaceae**	37	1.88	0.061	0.20	-0.01	0.41
41	Crassulaceae	2	-1.15	0.250	0.00	-0.01	0.00
42	Cucurbitaceae	7	-2.16	0.031	-0.03	-0.06	0.00
43	Cunoniaceae	4	-1.00	0.317	-0.01	-0.03	0.01

44	Cyperaceae	3	-1.69	0.092	-0.01	-0.03	0.00
45	Dilleniaceae	3	-1.55	0.120	-0.02	-0.04	0.00
46	Ebenaceae	1	-1.00	0.317	0.00	0.00	0.00
47	Ericaceae	2	1.44	0.149	0.08	-0.03	0.19
48	Eriocaulaceae	1	-1.00	0.317	-0.01	-0.03	0.01
49	Erythroxylaceae	6	-1.86	0.063	-0.03	-0.07	0.00
50	**Euphorbiaceae**	28	-2.11	0.035	-0.07	-0.14	-0.01
51	**Fabaceae**	232	-0.23	0.817	-0.04	-0.35	0.28
52	Geraniaceae	2	-1.14	0.254	-0.01	-0.01	0.00
53	Humiriaceae	1	-1.00	0.317	-0.01	-0.03	0.01
54	Hypericaceae	1	-1.00	0.317	0.00	-0.01	0.00
55	Iridaceae	8	-2.54	0.011	-0.03	-0.06	-0.01
56	Krameriaceae	1	-1.00	0.317	0.00	-0.01	0.00
57	**Lamiaceae**	30	1.33	0.183	0.13	-0.06	0.33
58	Lauraceae	9	-2.63	0.009	-0.05	-0.08	-0.01
59	Lecythidaceae	4	-1.68	0.092	-0.02	-0.03	0.00
60	Liliaceae	1	-1.00	0.317	0.00	0.00	0.00
61	Linaceae	1	-1.00	0.317	0.00	-0.01	0.00
62	Loranthaceae	9	0.24	0.813	0.01	-0.05	0.06
63	**Lythraceae**	21	0.29	0.769	0.02	-0.12	0.17
64	Magnoliaceae	2	-1.38	0.167	-0.01	-0.02	0.00
65	**Malpighiaceae**	58	2.14	0.032	0.26	0.02	0.49
66	**Malvaceae**	57	1.72	0.085	0.15	-0.02	0.32
67	Marantaceae	2	-1.00	0.317	-0.01	-0.02	0.01
68	Marcgraviaceae	2	-1.00	0.317	-0.01	-0.02	0.01
69	**Melastomataceae**	39	-1.56	0.120	-0.12	-0.28	0.03
70	Meliaceae	3	-1.37	0.172	-0.01	-0.02	0.00
71	Menyanthaceae	1	-1.00	0.317	-0.01	-0.02	0.01
72	Monimiaceae	1	-1.00	0.317	0.00	-0.01	0.00
73	Moraceae	5	-2.00	0.046	-0.03	-0.06	0.00
74	Muntingiaceae	1	-1.00	0.317	0.00	0.00	0.00
75	**Myrtaceae**	54	-0.73	0.463	-0.05	-0.20	0.09
76	Nyctaginaceae	9	-2.08	0.037	-0.04	-0.07	0.00
77	Nymphaeaceae	2	-1.00	0.317	0.00	0.00	0.00
78	Ochnaceae	8	2.37	0.018	0.17	0.03	0.31
79	Olacaceae	1	-1.00	0.317	0.00	-0.01	0.00
80	Oleaceae	2	-1.14	0.254	-0.01	-0.01	0.00
81	Onagraceae	8	0.49	0.623	0.02	-0.05	0.08
82	Orchidaceae	1	-1.00	0.317	-0.01	-0.02	0.01
83	**Oxalidaceae**	13	-0.28	0.783	-0.01	-0.11	0.08
84	**Passifloraceae**	26	-1.50	0.134	-0.07	-0.17	0.02
85	Phytolaccaceae	4	-1.92	0.054	-0.01	-0.02	0.00
86	Plantaginaceae	3	-1.50	0.135	-0.01	-0.02	0.00
87	Plumbaginaceae	3	-1.76	0.079	-0.01	-0.02	0.00

88	**Poaceae**	12	-1.74	0.081	-0.05	-0.10	0.01
89	Polygalaceae	4	-1.97	0.048	-0.02	-0.04	0.00
90	**Polygonaceae**	11	-3.09	0.002	-0.03	-0.05	-0.01
91	Portulacaceae	3	1.40	0.163	0.07	-0.03	0.17
92	Primulaceae	5	0.49	0.624	0.02	-0.05	0.09
93	Proteaceae	8	-2.84	0.004	-0.03	-0.05	-0.01
94	Ranunculaceae	2	-1.40	0.161	-0.01	-0.02	0.00
95	Rhamnaceae	5	-2.26	0.024	-0.02	-0.03	0.00
96	Rosaceae	8	-2.09	0.036	-0.02	-0.04	0.00
97	**Rubiaceae**	68	-0.30	0.767	-0.04	-0.27	0.20
98	Rutaceae	4	-1.84	0.066	-0.02	-0.04	0.00
99	Salicaceae	3	0.88	0.381	0.03	-0.04	0.10
100	**Sapindaceae**	23	1.80	0.072	0.16	-0.01	0.33
101	Sapotaceae	5	-2.11	0.035	-0.03	-0.06	0.00
102	Scrophulariaceae	1	-1.00	0.317	0.00	-0.01	0.00
103	Smilacaceae	1	-1.00	0.317	0.00	-0.01	0.00
104	**Solanaceae**	47	1.07	0.283	0.09	-0.08	0.26
105	Strelitziaceae	2	-1.15	0.250	0.00	-0.01	0.00
106	Styracaceae	5	0.49	0.621	0.01	-0.04	0.07
107	Symplocaceae	2	0.68	0.498	0.02	-0.05	0.09
108	Talinaceae	1	-1.00	0.317	0.00	-0.01	0.00
109	Theaceae	5	-1.59	0.112	-0.01	-0.03	0.00
110	Thymelaeaceae	3	-1.63	0.104	-0.02	-0.05	0.00
111	Tropaeolaceae	1	-1.00	0.317	0.00	0.00	0.00
112	Urticaceae	2	-1.41	0.159	-0.01	-0.02	0.00
113	Velloziaceae	3	-1.03	0.301	-0.02	-0.06	0.02
114	**Verbenaceae**	27	0.42	0.675	0.02	-0.08	0.13
115	Vitaceae	1	-1.00	0.317	0.00	-0.01	0.00
116	Vochysiaceae	8	0.85	0.396	0.04	-0.05	0.12
117	Winteraceae	1	-1.00	0.317	0.00	-0.01	0.00
118	Xanthorrhoeaceae	3	-1.27	0.205	-0.01	-0.01	0.00
119	Ximeniaceae	1	-1.00	0.317	0.00	-0.01	0.00
120	Xyridaceae	1	-1.00	0.317	-0.01	-0.03	0.01
121	Zygophyllaceae	1	-1.00	0.317	-0.01	-0.03	0.01

Table S5. Results from *z*-score analyses of expected and observed selection of 121 families in bee-plant networks for recovery of bee visitation rates. Common families (at least 10 species

occurrences) are listed in bold. Families with confidence intervals that do not overlap zero selected significantly more/less than expected by chance.

No.	Family	Occ.	*z*-score	*P*	Ave. diff.	-95% CI	+95% CI
1	**Acanthaceae**	14	-0.41	0.681	-0.02	-0.12	0.08
2	Adoxaceae	1	-1.00	0.317	-0.01	-0.03	0.01
3	Aizoaceae	1	1.00	0.317	0.03	-0.03	0.10
4	Alismataceae	2	0.07	0.947	0.00	-0.08	0.09
5	Amaranthaceae	8	-1.25	0.211	-0.06	-0.16	0.04
6	Amaryllidaceae	1	-1.00	0.317	-0.01	-0.03	0.01
7	**Anacardiaceae**	26	0.74	0.458	0.08	-0.13	0.29
8	Annonaceae	1	NA	NA	0.00	NA	NA
9	Apiaceae	3	0.18	0.858	0.01	-0.07	0.08
10	**Apocynaceae**	22	-3.60	0.000	-0.16	-0.25	-0.07
11	Aquifoliaceae	5	1.77	0.076	0.07	-0.01	0.14
12	Araliaceae	7	1.03	0.305	0.04	-0.04	0.13
13	Arecaceae	9	0.54	0.590	0.03	-0.07	0.12
14	Asparagaceae	7	1.43	0.152	0.05	-0.02	0.13
15	**Asteraceae**	182	-2.12	0.034	-0.32	-0.61	-0.02
16	Balsaminaceae	3	0.73	0.467	0.05	-0.08	0.18
17	Begoniaceae	6	-1.00	0.317	-0.10	-0.28	0.09
18	**Bignoniaceae**	41	-1.63	0.102	-0.17	-0.37	0.03
19	Bixaceae	3	0.26	0.797	0.01	-0.06	0.07
20	**Boraginaceae**	16	0.67	0.502	0.06	-0.12	0.24
21	Brassicaceae	3	0.77	0.439	0.05	-0.07	0.16
22	Bromeliaceae	5	0.31	0.754	0.01	-0.08	0.11
23	Burseraceae	2	-1.00	0.317	-0.01	-0.04	0.01
24	Cactaceae	7	-1.32	0.188	-0.04	-0.11	0.02
25	Calophyllaceae	3	-0.62	0.533	-0.02	-0.10	0.05
26	Campanulaceae	1	-1.00	0.317	-0.02	-0.05	0.02
27	Capparaceae	4	-0.11	0.909	0.00	-0.05	0.04
28	Caprifoliaceae	2	0.25	0.803	0.01	-0.06	0.08
29	Caryocaraceae	4	-0.07	0.944	0.00	-0.08	0.07
30	Caryophyllaceae	2	-1.43	0.154	-0.02	-0.05	0.01
31	Celastraceae	1	1.00	0.317	0.03	-0.03	0.10
32	Chloranthaceae	1	NA	NA	0.00	NA	NA
33	Chrysobalanaceae	5	0.59	0.553	0.03	-0.07	0.13
34	Cleomaceae	2	-1.76	0.079	-0.04	-0.08	0.00
35	Clethraceae	1	NA	NA	0.00	NA	NA
36	Clusiaceae	3	-0.27	0.789	-0.01	-0.07	0.05
37	Combretaceae	4	0.51	0.611	0.03	-0.07	0.12
38	Commelinaceae	6	-2.42	0.016	-0.06	-0.11	-0.01

39	Connaraceae	1	-1.00	0.317	-0.01	-0.03	0.01
40	**Convolvulaceae**	37	-0.41	0.682	-0.03	-0.17	0.11
41	Crassulaceae	2	-1.44	0.151	-0.02	-0.05	0.01
42	Cucurbitaceae	7	-0.72	0.473	-0.04	-0.14	0.06
43	Cunoniaceae	4	-2.43	0.015	-0.06	-0.11	-0.01
44	Cyperaceae	3	-0.66	0.512	-0.02	-0.09	0.04
45	Dilleniaceae	3	-1.80	0.072	-0.03	-0.07	0.00
46	Ebenaceae	1	-1.00	0.317	-0.01	-0.03	0.01
47	Ericaceae	2	1.43	0.151	0.06	-0.02	0.14
48	Eriocaulaceae	1	-1.00	0.317	-0.01	-0.04	0.01
49	Erythroxylaceae	6	-0.89	0.375	-0.04	-0.14	0.05
50	**Euphorbiaceae**	28	-1.37	0.169	-0.13	-0.31	0.05
51	**Fabaceae**	232	0.23	0.817	0.07	-0.51	0.64
52	Geraniaceae	2	-1.43	0.154	-0.02	-0.05	0.01
53	Humiriaceae	1	1.44	0.149	0.05	-0.02	0.13
54	Hypericaceae	1	1.00	0.317	0.03	-0.03	0.09
55	Iridaceae	8	-2.72	0.007	-0.09	-0.15	-0.02
56	Krameriaceae	1	1.00	0.317	0.03	-0.03	0.10
57	**Lamiaceae**	30	1.61	0.107	0.18	-0.04	0.40
58	Lauraceae	9	1.30	0.195	0.09	-0.05	0.22
59	Lecythidaceae	4	-1.60	0.109	-0.04	-0.10	0.01
60	Liliaceae	1	0.66	0.507	0.02	-0.04	0.08
61	Linaceae	1	-1.00	0.317	-0.01	-0.04	0.01
62	Loranthaceae	9	-2.36	0.018	-0.05	-0.09	-0.01
63	**Lythraceae**	21	2.08	0.038	0.18	0.01	0.35
64	Magnoliaceae	2	NA	NA	0.00	NA	NA
65	**Malpighiaceae**	58	1.05	0.296	0.16	-0.14	0.47
66	**Malvaceae**	57	0.52	0.605	0.08	-0.22	0.38
67	Marantaceae	2	-1.00	0.317	-0.02	-0.05	0.02
68	Marcgraviaceae	2	1.00	0.317	0.03	-0.03	0.09
69	**Melastomataceae**	39	0.31	0.757	0.05	-0.27	0.38
70	Meliaceae	3	-2.08	0.038	-0.05	-0.09	0.00
71	Menyanthaceae	1	-1.00	0.317	-0.01	-0.04	0.01
72	Monimiaceae	1	NA	NA	0.00	NA	NA
73	Moraceae	5	-2.21	0.027	-0.07	-0.14	-0.01
74	Muntingiaceae	1	-1.44	0.151	-0.02	-0.05	0.01
75	**Myrtaceae**	54	0.51	0.609	0.07	-0.19	0.33
76	Nyctaginaceae	9	-1.58	0.114	-0.08	-0.19	0.02
77	Nymphaeaceae	2	-1.44	0.149	-0.03	-0.08	0.01
78	Ochnaceae	8	1.29	0.197	0.08	-0.04	0.20
79	Olacaceae	1	-1.00	0.317	-0.02	-0.05	0.02
80	Oleaceae	2	-1.43	0.154	-0.02	-0.05	0.01
81	Onagraceae	8	-1.02	0.306	-0.02	-0.07	0.02
82	Orchidaceae	1	-2.11	0.035	-0.05	-0.10	0.00

83	**Oxalidaceae**	13	0.23	0.816	0.01	-0.08	0.10
84	**Passifloraceae**	26	-1.02	0.308	-0.09	-0.26	0.08
85	Phytolaccaceae	4	-2.37	0.018	-0.06	-0.10	-0.01
86	Plantaginaceae	3	-0.39	0.697	-0.01	-0.08	0.05
87	Plumbaginaceae	3	-1.00	0.317	-0.01	-0.03	0.01
88	**Poaceae**	12	-0.90	0.370	-0.04	-0.13	0.05
89	Polygalaceae	4	-0.63	0.527	-0.03	-0.10	0.05
90	**Polygonaceae**	11	0.64	0.521	0.03	-0.07	0.13
91	Portulacaceae	3	1.44	0.150	0.06	-0.02	0.14
92	Primulaceae	5	0.60	0.546	0.03	-0.08	0.15
93	Proteaceae	8	0.74	0.458	0.03	-0.05	0.12
94	Ranunculaceae	2	-1.00	0.317	-0.01	-0.04	0.01
95	Rhamnaceae	5	1.27	0.205	0.06	-0.03	0.14
96	Rosaceae	8	0.44	0.658	0.02	-0.07	0.11
97	**Rubiaceae**	68	0.12	0.905	0.02	-0.28	0.32
98	Rutaceae	4	-1.44	0.151	-0.03	-0.07	0.01
99	Salicaceae	3	0.01	0.990	0.00	-0.03	0.03
100	**Sapindaceae**	23	1.49	0.137	0.10	-0.03	0.23
101	Sapotaceae	5	-1.08	0.279	-0.05	-0.13	0.04
102	Scrophulariaceae	1	-1.00	0.317	-0.01	-0.04	0.01
103	Smilacaceae	1	NA	NA	0.00	NA	NA
104	**Solanaceae**	47	1.62	0.106	0.16	-0.04	0.36
105	Strelitziaceae	2	-1.45	0.148	-0.02	-0.04	0.01
106	Styracaceae	5	-0.88	0.378	-0.03	-0.08	0.03
107	Symplocaceae	2	1.44	0.149	0.05	-0.02	0.13
108	Talinaceae	1	-1.00	0.317	-0.01	-0.02	0.01
109	Theaceae	5	0.56	0.578	0.02	-0.06	0.11
110	Thymelaeaceae	3	0.98	0.325	0.04	-0.04	0.11
111	Tropaeolaceae	1	-1.00	0.317	-0.01	-0.03	0.01
112	Urticaceae	2	-1.00	0.317	-0.01	-0.02	0.01
113	Velloziaceae	3	0.38	0.706	0.01	-0.03	0.04
114	**Verbenaceae**	27	-1.05	0.294	-0.09	-0.26	0.08
115	Vitaceae	1	0.60	0.545	0.02	-0.05	0.09
116	Vochysiaceae	8	1.45	0.148	0.08	-0.03	0.19
117	Winteraceae	1	0.58	0.561	0.02	-0.04	0.08
118	Xanthorrhoeaceae	3	0.45	0.656	0.01	-0.04	0.06
119	Ximeniaceae	1	0.44	0.658	0.02	-0.06	0.09
120	Xyridaceae	1	0.17	0.861	0.01	-0.05	0.06
121	Zygophyllaceae	1	-1.00	0.317	-0.01	-0.03	0.01

Table S6. Results from *z*-score analyses of expected and observed selection of 121 families in bee-plant networks for recovery of native bee richness (excluding visits by non-native honeybees *Apis mellifera*). Common families (at least 10 species occurrences) are listed in bold. Families with confidence intervals that do not overlap zero selected significantly more/less than expected by chance.

No.	Family	Occ.	*z*-score	*P*	Ave. diff.	-95% CI	+95% CI
1	**Acanthaceae**	14	0.09	0.928	0.00	-0.06	0.07
2	Adoxaceae	1	-1.00	0.317	0.00	0.00	0.00
3	Aizoaceae	1	-1.00	0.317	0.00	0.00	0.00
4	Alismataceae	2	-1.00	0.317	-0.01	-0.04	0.01
5	Amaranthaceae	8	-2.51	0.012	-0.03	-0.05	-0.01
6	Amaryllidaceae	1	-1.00	0.317	0.00	0.00	0.00
7	**Anacardiaceae**	26	-4.49	0.000	-0.11	-0.15	-0.06
8	Annonaceae	1	-1.00	0.317	0.00	-0.01	0.00
9	Apiaceae	3	-1.44	0.150	-0.01	-0.03	0.00
10	**Apocynaceae**	22	-0.83	0.405	-0.04	-0.12	0.05
11	Aquifoliaceae	5	0.22	0.824	0.01	-0.07	0.09
12	Araliaceae	7	1.35	0.176	0.09	-0.04	0.21
13	Arecaceae	9	0.24	0.813	0.01	-0.07	0.09
14	Asparagaceae	7	-1.40	0.162	-0.02	-0.04	0.01
15	**Asteraceae**	184	0.34	0.734	0.05	-0.25	0.36
16	Balsaminaceae	3	-1.39	0.164	0.00	-0.01	0.00
17	Begoniaceae	6	-1.00	0.317	-0.02	-0.05	0.02
18	**Bignoniaceae**	41	-0.18	0.860	-0.01	-0.17	0.15
19	Bixaceae	3	-1.76	0.079	-0.01	-0.02	0.00
20	**Boraginaceae**	16	-0.52	0.604	-0.02	-0.10	0.06
21	Brassicaceae	3	0.87	0.385	0.03	-0.04	0.09
22	Bromeliaceae	5	-2.30	0.021	-0.02	-0.04	0.00
23	Burseraceae	2	-1.42	0.155	-0.01	-0.02	0.00
24	Cactaceae	7	-1.75	0.080	-0.03	-0.07	0.00
25	Calophyllaceae	3	0.56	0.576	0.01	-0.03	0.06
26	Campanulaceae	1	-1.00	0.317	-0.01	-0.02	0.01
27	Capparaceae	4	-1.36	0.175	-0.02	-0.04	0.01
28	Caprifoliaceae	2	-1.21	0.225	0.00	-0.01	0.00
29	Caryocaraceae	4	-2.07	0.039	-0.02	-0.04	0.00
30	Caryophyllaceae	2	-1.14	0.255	-0.01	-0.01	0.00
31	Celastraceae	1	-1.00	0.317	0.00	-0.01	0.00
32	Chloranthaceae	1	-1.00	0.317	0.00	-0.01	0.00
33	Chrysobalanaceae	5	0.64	0.524	0.02	-0.05	0.10

34	Cleomaceae	2	-1.14	0.256	-0.01	-0.01	0.00
35	Clethraceae	1	-1.00	0.317	0.00	-0.01	0.00
36	Clusiaceae	3	-1.43	0.154	-0.01	-0.02	0.00
37	Combretaceae	4	-2.13	0.033	-0.02	-0.03	0.00
38	Commelinaceae	6	-2.33	0.020	-0.02	-0.04	0.00
39	Connaraceae	1	-1.00	0.317	0.00	-0.01	0.00
40	**Convolvulaceae**	37	1.87	0.062	0.20	-0.01	0.41
41	Crassulaceae	2	-1.15	0.251	0.00	-0.01	0.00
42	Cucurbitaceae	7	-2.16	0.031	-0.03	-0.06	0.00
43	Cunoniaceae	4	-1.00	0.317	-0.01	-0.03	0.01
44	Cyperaceae	3	-1.69	0.092	-0.01	-0.03	0.00
45	Dilleniaceae	3	-1.55	0.120	-0.02	-0.04	0.00
46	Ebenaceae	1	-1.00	0.317	0.00	0.00	0.00
47	Ericaceae	2	0.89	0.373	0.04	-0.04	0.12
48	Eriocaulaceae	1	1.00	0.317	0.03	-0.03	0.09
49	Erythroxylaceae	6	-1.86	0.063	-0.03	-0.07	0.00
50	**Euphorbiaceae**	28	-2.12	0.034	-0.07	-0.14	-0.01
51	**Fabaceae**	228	-0.17	0.867	-0.03	-0.36	0.31
52	Geraniaceae	2	-1.14	0.255	-0.01	-0.01	0.00
53	Humiriaceae	1	-1.00	0.317	-0.01	-0.03	0.01
54	Hypericaceae	1	-1.00	0.317	0.00	-0.01	0.00
55	Iridaceae	8	-2.54	0.011	-0.03	-0.06	-0.01
56	Krameriaceae	1	-1.00	0.317	0.00	-0.01	0.00
57	**Lamiaceae**	31	0.16	0.873	0.01	-0.09	0.10
58	Lauraceae	9	-2.63	0.009	-0.05	-0.08	-0.01
59	Lecythidaceae	4	-1.68	0.092	-0.02	-0.03	0.00
60	Liliaceae	1	-1.00	0.317	0.00	0.00	0.00
61	Linaceae	1	-1.00	0.317	0.00	-0.01	0.00
62	Loranthaceae	9	0.24	0.813	0.01	-0.05	0.06
63	**Lythraceae**	21	0.29	0.770	0.02	-0.12	0.17
64	Magnoliaceae	2	-1.38	0.167	-0.01	-0.02	0.00
65	**Malpighiaceae**	58	2.18	0.029	0.17	0.02	0.33
66	**Malvaceae**	57	1.53	0.125	0.15	-0.04	0.34
67	Marantaceae	2	-1.00	0.317	-0.01	-0.02	0.01
68	Marcgraviaceae	2	-1.00	0.317	-0.01	-0.02	0.01
69	**Melastomataceae**	39	0.07	0.943	0.00	-0.09	0.09
70	Meliaceae	3	-1.37	0.170	-0.01	-0.02	0.00
71	Menyanthaceae	1	-1.00	0.317	-0.01	-0.02	0.01
72	Monimiaceae	1	-1.00	0.317	0.00	-0.01	0.00
73	Moraceae	5	-2.00	0.046	-0.03	-0.06	0.00
74	Muntingiaceae	1	-1.00	0.317	0.00	0.00	0.00
75	**Myrtaceae**	54	-0.73	0.463	-0.05	-0.20	0.09
76	Nyctaginaceae	9	-2.08	0.037	-0.04	-0.07	0.00
77	Nymphaeaceae	2	-1.00	0.317	0.00	0.00	0.00

78	Ochnaceae	8	2.37	0.018	0.17	0.03	0.31
79	Olacaceae	1	-1.00	0.317	0.00	-0.01	0.00
80	Oleaceae	2	-1.14	0.255	-0.01	-0.01	0.00
81	Onagraceae	8	0.49	0.623	0.02	-0.05	0.08
82	Orchidaceae	1	-1.00	0.317	-0.01	-0.02	0.01
83	**Oxalidaceae**	12	-0.28	0.782	-0.01	-0.11	0.08
84	**Passifloraceae**	26	0.59	0.556	0.05	-0.12	0.22
85	Phytolaccaceae	4	-1.92	0.054	-0.01	-0.02	0.00
86	Plantaginaceae	3	-1.50	0.135	-0.01	-0.02	0.00
87	Plumbaginaceae	3	-1.76	0.079	-0.01	-0.02	0.00
88	**Poaceae**	12	-1.74	0.081	-0.05	-0.10	0.01
89	Polygalaceae	4	-1.97	0.048	-0.02	-0.04	0.00
90	**Polygonaceae**	11	0.20	0.838	0.01	-0.07	0.09
91	Portulacaceae	3	0.77	0.440	0.03	-0.05	0.11
92	Primulaceae	5	0.49	0.624	0.02	-0.05	0.09
93	Proteaceae	8	-2.84	0.004	-0.03	-0.05	-0.01
94	Ranunculaceae	2	-1.40	0.161	-0.01	-0.02	0.00
95	Rhamnaceae	5	-2.27	0.024	-0.02	-0.03	0.00
96	Rosaceae	8	-2.09	0.036	-0.02	-0.04	0.00
97	**Rubiaceae**	70	0.05	0.962	0.01	-0.22	0.23
98	Rutaceae	4	-1.84	0.066	-0.02	-0.04	0.00
99	Salicaceae	3	0.88	0.381	0.03	-0.04	0.10
100	**Sapindaceae**	23	-1.11	0.265	-0.05	-0.15	0.04
101	Sapotaceae	5	-2.11	0.034	-0.03	-0.06	0.00
102	Scrophulariaceae	1	-1.00	0.317	0.00	-0.01	0.00
103	Smilacaceae	1	-1.00	0.317	0.00	-0.01	0.00
104	**Solanaceae**	47	1.21	0.227	0.09	-0.06	0.24
105	Strelitziaceae	2	-1.15	0.251	0.00	-0.01	0.00
106	Styracaceae	5	0.49	0.621	0.01	-0.04	0.07
107	Symplocaceae	2	0.68	0.498	0.02	-0.05	0.09
108	Talinaceae	1	-1.00	0.317	0.00	-0.01	0.00
109	Theaceae	5	-1.59	0.112	-0.01	-0.03	0.00
110	Thymelaeaceae	3	-1.63	0.104	-0.02	-0.05	0.00
111	Tropaeolaceae	1	-1.00	0.317	0.00	0.00	0.00
112	Urticaceae	2	-1.41	0.159	-0.01	-0.02	0.00
113	Velloziaceae	3	-1.03	0.301	-0.02	-0.06	0.02
114	**Verbenaceae**	27	0.42	0.676	0.02	-0.08	0.13
115	Vitaceae	1	-1.00	0.317	0.00	-0.01	0.00
116	Vochysiaceae	8	0.85	0.396	0.04	-0.05	0.12
117	Winteraceae	1	-1.00	0.317	0.00	-0.01	0.00
118	Xanthorrhoeaceae	3	-1.27	0.205	-0.01	-0.01	0.00
119	Ximeniaceae	1	-1.00	0.317	0.00	-0.01	0.00
120	Xyridaceae	1	-1.00	0.317	-0.01	-0.03	0.01
121	Zygophyllaceae	1	-1.00	0.317	-0.01	-0.03	0.01

Table S7. Results from *z*-score analyses of expected and observed selection of 121 families in bee-plant networks for recovery of native bee visitation rates (excluding visits by non-native honeybees *Apis mellifera*). Common families (at least 10 species occurrences) are listed in bold. Families with confidence intervals that do not overlap zero selected significantly more/less than expected by chance.

No.	Family	Occ.	*z*-score	*P*	Ave. diff.	-95% CI	+95% CI
1	**Acanthaceae**	14	0.34	0.732	0.05	-0.22	0.32
2	Adoxaceae	1	-1.00	0.317	-0.01	-0.02	0.01
3	Aizoaceae	1	1.00	0.317	0.03	-0.03	0.10
4	Alismataceae	2	-1.00	0.317	-0.02	-0.07	0.02
5	Amaranthaceae	8	-0.07	0.944	0.00	-0.11	0.10
6	Amaryllidaceae	1	-1.00	0.317	-0.01	-0.02	0.01
7	**Anacardiaceae**	26	-0.55	0.585	-0.04	-0.20	0.11
8	Annonaceae	1	-1.00	0.317	-0.02	-0.04	0.01
9	Apiaceae	3	0.24	0.812	0.01	-0.07	0.09
10	**Apocynaceae**	22	-4.08	0.000	-0.16	-0.23	-0.08
11	Aquifoliaceae	5	-0.64	0.522	-0.03	-0.12	0.06
12	Araliaceae	7	2.11	0.035	0.16	0.01	0.31
13	Arecaceae	9	0.94	0.347	0.06	-0.07	0.19
14	Asparagaceae	7	0.81	0.417	0.03	-0.05	0.11
15	**Asteraceae**	184	-1.45	0.147	-0.28	-0.66	0.10
16	Balsaminaceae	3	0.72	0.474	0.05	-0.09	0.19
17	Begoniaceae	6	-1.00	0.317	-0.09	-0.27	0.09
18	**Bignoniaceae**	41	-0.18	0.854	-0.02	-0.21	0.18
19	Bixaceae	3	-2.14	0.033	-0.04	-0.07	0.00
20	**Boraginaceae**	16	-0.13	0.895	-0.01	-0.17	0.15
21	Brassicaceae	3	0.77	0.439	0.05	-0.07	0.16
22	Bromeliaceae	5	-0.37	0.715	-0.01	-0.09	0.06
23	Burseraceae	2	-1.42	0.155	-0.02	-0.05	0.01
24	Cactaceae	7	0.09	0.927	0.00	-0.07	0.08
25	Calophyllaceae	3	-0.04	0.966	0.00	-0.07	0.07
26	Campanulaceae	1	-1.00	0.317	-0.02	-0.05	0.02
27	Capparaceae	4	0.16	0.872	0.00	-0.04	0.05
28	Caprifoliaceae	2	-1.35	0.177	-0.02	-0.05	0.01
29	Caryocaraceae	4	-0.01	0.989	0.00	-0.08	0.08

30	Caryophyllaceae	2	-1.39	0.165	-0.02	-0.05	0.01
31	Celastraceae	1	-1.00	0.317	-0.01	-0.03	0.01
32	Chloranthaceae	1	1.00	0.317	0.03	-0.03	0.08
33	Chrysobalanaceae	5	0.65	0.515	0.03	-0.07	0.13
34	Cleomaceae	2	-1.40	0.160	-0.02	-0.04	0.01
35	Clethraceae	1	1.00	0.317	0.03	-0.03	0.08
36	Clusiaceae	3	-1.73	0.084	-0.04	-0.07	0.00
37	Combretaceae	4	-0.09	0.928	0.00	-0.07	0.07
38	Commelinaceae	6	-2.10	0.036	-0.07	-0.14	0.00
39	Connaraceae	1	-1.00	0.317	-0.01	-0.03	0.01
40	**Convolvulaceae**	37	-0.57	0.569	-0.05	-0.22	0.12
41	Crassulaceae	2	-1.00	0.317	-0.01	-0.02	0.01
42	Cucurbitaceae	7	-2.69	0.007	-0.09	-0.16	-0.03
43	Cunoniaceae	4	-1.00	0.317	-0.02	-0.06	0.02
44	Cyperaceae	3	-1.76	0.078	-0.03	-0.06	0.00
45	Dilleniaceae	3	-1.76	0.078	-0.03	-0.06	0.00
46	Ebenaceae	1	-1.00	0.317	-0.01	-0.02	0.01
47	Ericaceae	2	1.44	0.150	0.08	-0.03	0.18
48	Eriocaulaceae	1	-1.00	0.317	-0.01	-0.04	0.01
49	Erythroxylaceae	6	-1.87	0.062	-0.07	-0.15	0.00
50	**Euphorbiaceae**	28	-0.15	0.878	-0.02	-0.22	0.19
51	**Fabaceae**	232	-0.17	0.865	-0.04	-0.57	0.48
52	Geraniaceae	2	-1.39	0.165	-0.02	-0.05	0.01
53	Humiriaceae	1	-1.00	0.317	-0.01	-0.04	0.01
54	Hypericaceae	1	-1.00	0.317	-0.01	-0.02	0.01
55	Iridaceae	8	-1.10	0.272	-0.04	-0.11	0.03
56	Krameriaceae	1	1.00	0.317	0.03	-0.03	0.10
57	**Lamiaceae**	30	1.61	0.108	0.12	-0.03	0.27
58	Lauraceae	9	-0.83	0.409	-0.04	-0.14	0.06
59	Lecythidaceae	4	-1.66	0.097	-0.04	-0.09	0.01
60	Liliaceae	1	-1.00	0.317	-0.01	-0.02	0.01
61	Linaceae	1	-1.00	0.317	-0.01	-0.04	0.01
62	Loranthaceae	9	-2.07	0.039	-0.04	-0.08	0.00
63	**Lythraceae**	21	1.28	0.201	0.13	-0.07	0.33
64	Magnoliaceae	2	-1.45	0.148	-0.02	-0.04	0.01
65	**Malpighiaceae**	58	2.64	0.008	0.43	0.11	0.74
66	**Malvaceae**	57	1.52	0.129	0.17	-0.05	0.40
67	Marantaceae	2	-1.00	0.317	-0.03	-0.09	0.03
68	Marcgraviaceae	2	-1.00	0.317	-0.03	-0.09	0.03
69	**Melastomataceae**	39	0.93	0.352	0.11	-0.12	0.33
70	Meliaceae	3	-1.21	0.225	-0.04	-0.10	0.02
71	Menyanthaceae	1	-1.00	0.317	-0.01	-0.04	0.01
72	Monimiaceae	1	-1.00	0.317	-0.02	-0.04	0.01
73	Moraceae	5	-2.23	0.026	-0.06	-0.11	-0.01

74	Muntingiaceae	1	-1.00	0.317	-0.01	-0.02	0.01
75	**Myrtaceae**	54	-3.15	0.002	-0.30	-0.49	-0.11
76	Nyctaginaceae	9	-1.30	0.195	-0.06	-0.15	0.03
77	Nymphaeaceae	2	-1.00	0.317	-0.01	-0.04	0.01
78	Ochnaceae	8	1.45	0.148	0.09	-0.03	0.21
79	Olacaceae	1	-1.00	0.317	-0.02	-0.04	0.01
80	Oleaceae	2	-1.39	0.165	-0.02	-0.05	0.01
81	Onagraceae	8	0.32	0.745	0.01	-0.04	0.06
82	Orchidaceae	1	-1.00	0.317	-0.01	-0.04	0.01
83	**Oxalidaceae**	12	0.64	0.525	0.03	-0.06	0.11
84	**Passifloraceae**	26	1.20	0.231	0.13	-0.08	0.34
85	Phytolaccaceae	4	-2.01	0.044	-0.04	-0.08	0.00
86	Plantaginaceae	3	-2.09	0.036	-0.04	-0.07	0.00
87	Plumbaginaceae	3	0.01	0.992	0.00	-0.07	0.07
88	**Poaceae**	12	0.24	0.811	0.01	-0.09	0.12
89	Polygalaceae	4	-2.07	0.039	-0.05	-0.09	0.00
90	**Polygonaceae**	11	0.80	0.421	0.05	-0.07	0.16
91	Portulacaceae	3	0.97	0.332	0.05	-0.05	0.14
92	Primulaceae	5	1.32	0.188	0.05	-0.02	0.12
93	Proteaceae	8	-0.96	0.335	-0.04	-0.14	0.05
94	Ranunculaceae	2	-1.44	0.150	-0.03	-0.07	0.01
95	Rhamnaceae	5	0.61	0.540	0.03	-0.07	0.13
96	Rosaceae	8	-0.43	0.664	-0.01	-0.08	0.05
97	**Rubiaceae**	70	0.49	0.627	0.08	-0.25	0.42
98	Rutaceae	4	0.12	0.908	0.00	-0.06	0.07
99	Salicaceae	3	0.16	0.869	0.00	-0.03	0.03
100	**Sapindaceae**	23	0.52	0.606	0.05	-0.13	0.22
101	Sapotaceae	5	-2.22	0.026	-0.06	-0.12	-0.01
102	Scrophulariaceae	1	-1.00	0.317	-0.01	-0.04	0.01
103	Smilacaceae	1	-1.00	0.317	-0.01	-0.03	0.01
104	**Solanaceae**	47	1.94	0.052	0.19	0.00	0.39
105	Strelitziaceae	2	-1.00	0.317	-0.01	-0.02	0.01
106	Styracaceae	5	-0.47	0.636	-0.01	-0.06	0.04
107	Symplocaceae	2	0.43	0.664	0.01	-0.04	0.07
108	Talinaceae	1	-1.00	0.317	-0.01	-0.02	0.01
109	Theaceae	5	0.84	0.399	0.04	-0.06	0.14
110	Thymelaeaceae	3	0.13	0.895	0.00	-0.06	0.07
111	Tropaeolaceae	1	-1.00	0.317	-0.01	-0.02	0.01
112	Urticaceae	2	-1.41	0.159	-0.02	-0.05	0.01
113	Velloziaceae	3	0.51	0.611	0.01	-0.03	0.04
114	**Verbenaceae**	27	-0.29	0.768	-0.02	-0.16	0.12
115	Vitaceae	1	1.00	0.317	0.03	-0.03	0.10
116	Vochysiaceae	8	0.82	0.414	0.08	-0.11	0.27
117	Winteraceae	1	-1.00	0.317	-0.02	-0.04	0.01

118	Xanthorrhoeaceae	3	1.00	0.317	0.03	-0.03	0.08
119	Ximeniaceae	1	-1.00	0.317	-0.01	-0.02	0.01
120	Xyridaceae	1	-1.00	0.317	-0.01	-0.04	0.01
121	Zygophyllaceae	1	-1.00	0.317	-0.01	-0.04	0.01

Table S8. Information on 24 quantitative Brazilian bee-plant networks. Networks divided into two categories: SAV – 'Savannah-like' and FOR – 'Forest' biomes. Total duration (months) of data collection and time (days) between repeat surveys. For full citations see attached reference list.

Reference	Latitude (DMS)	Longitude (DMS)	Biome type	Duration (months)	Repeat surveys (days)	No. plant species	No. bee species
Aguiar 1995; Aguiar. & Martins 1997	7º25'S	36º30'W	SAV	12	15	37	45
Aguiar 2003; Aguiar & Zanella 2005	12º42'S	39º46'W	SAV	15	30	42	60
Albuquerque 1998	2º29' S	44º18' W	FOR	12	30	28	36
Andena 2005	22º15` S	47º W	SAV	12	15	56	103
Carvalho 1993	12º40' 39''S	39º06' 23''W	SAV	37	30	19	71
Carvalho 1999	12º45'S	39º26'W	SAV	14	15	61	80
D'Avila 2006	22º13'09"S	47º54'04"W	SAV	12	15	16	12
D'Avila 2006	22º13'09"S	47º54'04"W	SAV	12	15	19	18
Faria 1994	19º17' S	43º36' W	SAV	12	30	52	107
Faria-Mucci et al. 2003	20º28'22"S	43º33'50"W	SAV	12	30	42	72
Hoffmann 1990	30º05'00S	51º02'00W	FOR	29	15	95	140
Knoll 1990	23º33'S	46º43'W	FOR	12	10	132	14
Lima 2004	6º40' - 6º41'S	35º07' - 35º12'W	FOR	36	30	66	141
Mateus 1998	21°33' S	47°51' W	SAV	24	15	58	142
Pedro 1992	21º18' - 21º27' S	47º12' - 47º20' W	SAV	12	15	123	196
Rêgo 1998	4º5' S	43º30'W	SAV	12	30	26	41
Silva et al. 1999	7º3' 15''S	34º50' 52''W	FOR	13	15	23	36
Silva 2004	6º45' / 6º50''S	34º56'' / 35º05''W	FOR	24	30	47	40
Silveira 2006	07º08'S	34º51'W	FOR	12	15	45	79
Silveira 2006	07º06'S	34º51'W	FOR	12	15	42	59
Silveira 2006	07º11'S	34º48'W	FOR	12	15	29	79
Sofia 1996	23º22' S	51º10' W	FOR	12	15	72	66
Sofia 1996	21º11' S	47º48' W	SAV	12	15	75	28
Wilms 1995; Wilms et al. 1996	23º38'S	45º52'W	FOR	30	-	214	259

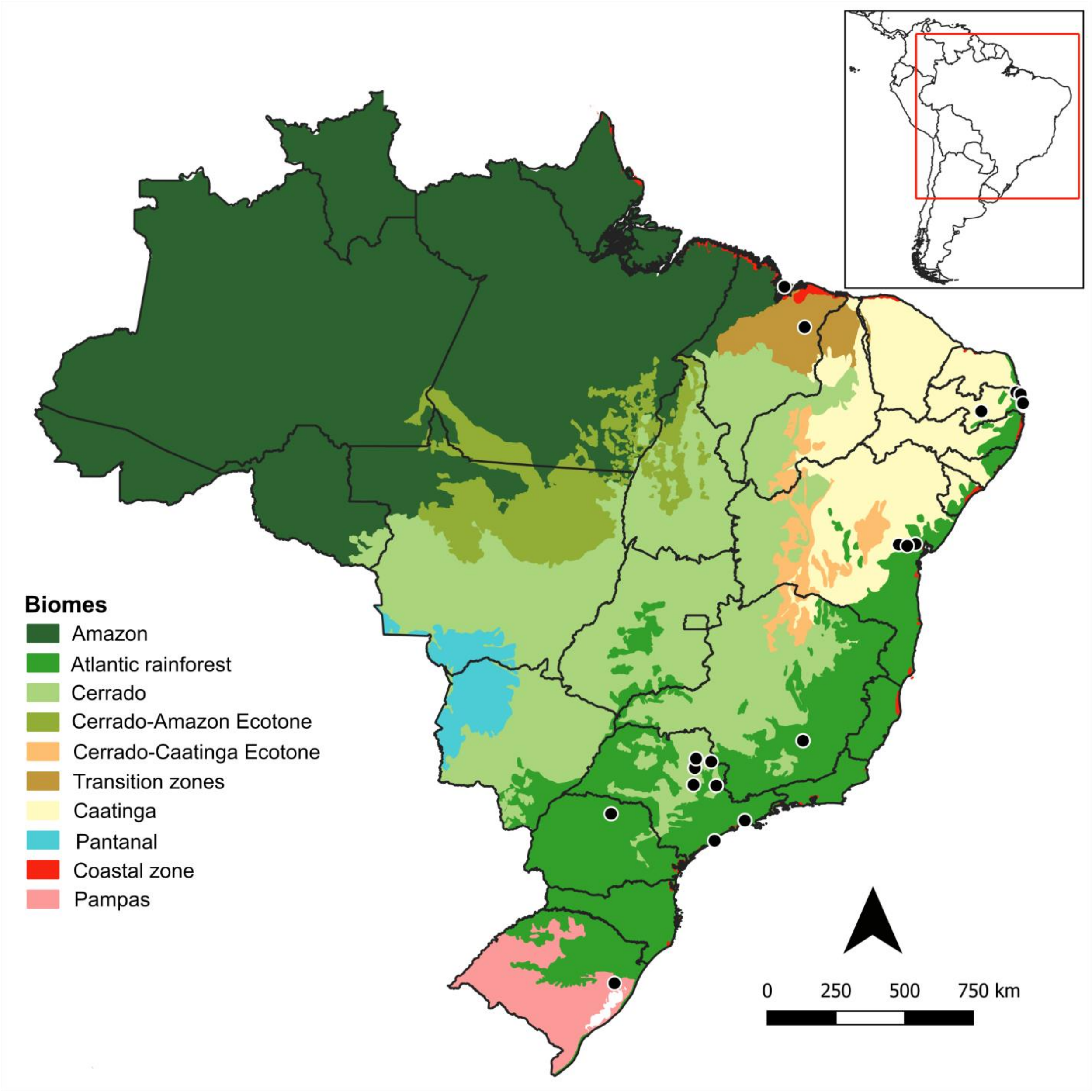

Figure S3. Brazilian biomes and locations of 24 sampled networks (source: Ministério do Meio Ambiente - http://mapas.mma.gov.br/i3geo/datadownload.htm).

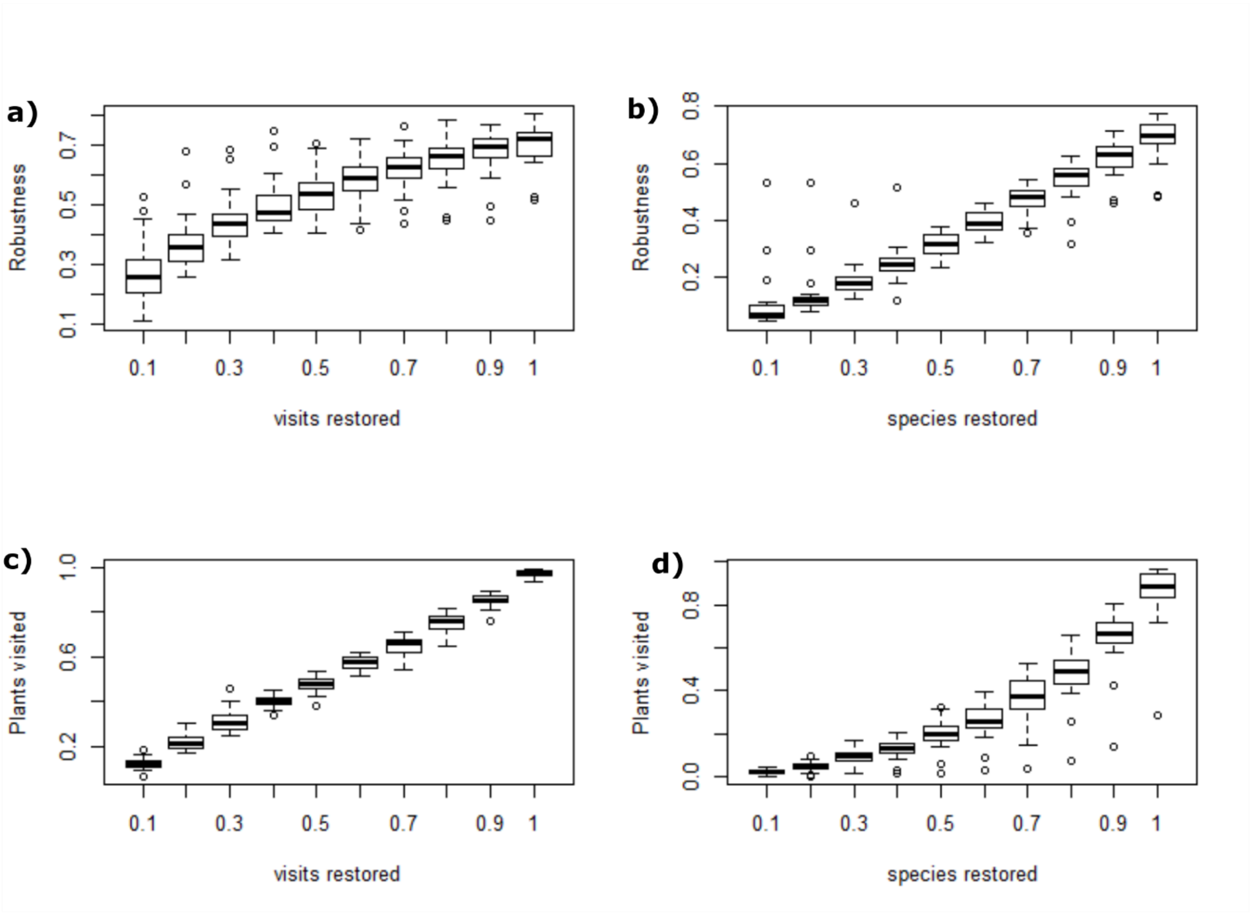

Figure S4. Boxplots to show robustness of restored networks to random extinction of higher-level (bee) species (a & b) and proportion of plant species with at least one bee flower visitor (c & d) under different restoration target thresholds (0.10 to 1.00 of proportion of total bee species/visits in pristine networks). Outer box shows interquartile range and thick horizontal line represents median values for 24 networks based on simulations (n = 30) of random subsets of full network containing different proportions (thresholds) of bee species and visits (300 simulations per network).

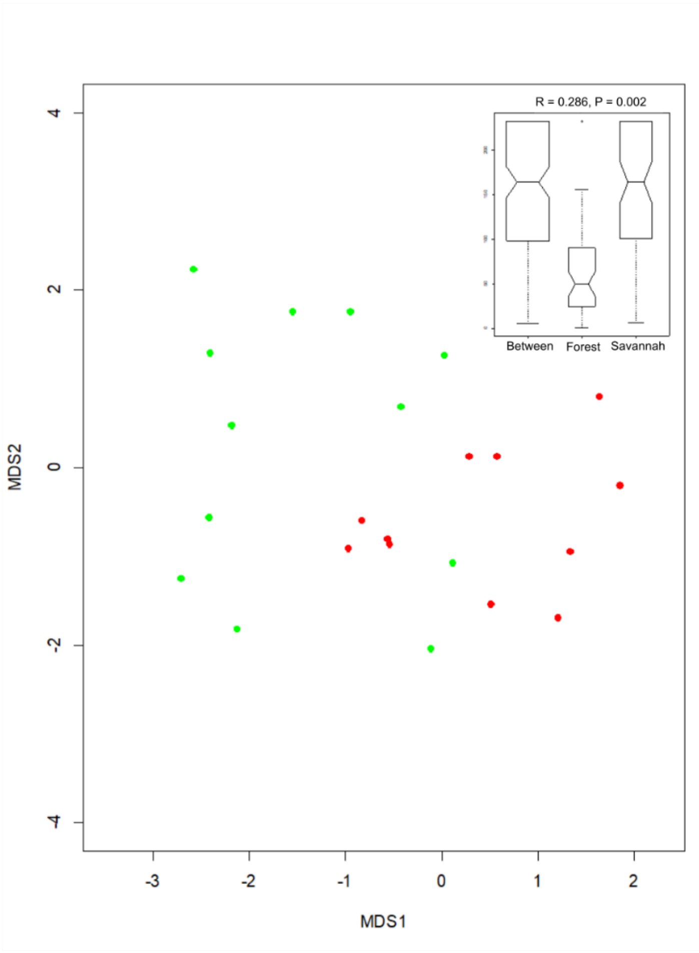

Figure S5. Non-metric Multi-dimensional Scaling (NMDS) ordination of plant communities from bee-plant networks belonging to two distinct biome types: ‘forest’ (red dots) and ‘savannah-like’ (green dots). Ordination analyses were weighted by plant abundance (insect visitation rates used as proxy). Inset panel shows results from analysis of similarity (ANOSIM).

Appendix I. Phylogenetic tree construction

To construct plant phylogenies of individual networks and the global species pool, we used the 'ComTreeOpt' function (Gastauer *et al.* 2018). This function builds highly resolved subfamily trees using phylogenetic relationships among species from the Open Tree of Life Project (Hinchliff *et al.* 2015), and inserts them into a previously defined 'supertree' containing relationships among all described angiosperm/plant families. We used phylogenetic tree 'R20160415.new' (Gastauer & Meira Neto 2017). Families containing species not recognized in the Open Tree of Life were inserted automatically, building politomies for genera within families and species within genera containing more than two species (Gastauer et al. 2018). Due to a high number of species in the global pool not recognized by the 'ComTreeOpt' function, this procedure was not viable for the entire species set. Therefore, we used the function 'tol_induced_subtree' to build genus trees for each family from our dataset; these were inserted into 'R20160415.new' via 'paste.tree' from the phytools R package (Revell 2012). Species were inserted into this genus tree using 'phylomatic' function from the Phylocom software (ver. 4.2, Webb & Donoghue 2005), pruning families not present in our dataset. All trees were dated using the 'bladj' function in combinations with age estimates for internal nodes from Magallón et al. (2015).

References

1. Aguiar, C.M.L. Abundância, diversidade e fenologia de abelhas (Hymenoptera - Apoidea) da caatinga (São João do Cariri, PB) e suas interações com a flora apícola. Msc Thesis, Universidade Federal da Paraíba, João Pessoa. (1995).

2. Aguiar, C.M.L. Utilização de recursos florais por abelhas (Hymenoptera: Apoidea) em uma área de Caatinga (Itatim, Bahia, Brasil). *Revista Brasileira de Zoologia*, **20**, 457-467. (2003).
3. Aguiar, C.M.L., & Martins, C.F. Abundância relativa, diversidade e fenologia de abelhas (Hymenoptera, Apoidea) na Caatinga, São João do Cariri, Paraíba, Brasil. *Iheringia*, **83**, 151-163. (1997).
4. Aguiar, C.M.L., & Zanella, F.C.V. Estrutura da comunidade de abelhas (Hymenoptera: Apoidea: Apiformis) de uma área na margem do domínio da Caatinga (Itatim, BA). *Neotropical Entomology*, **34**, 15-24. (2005).
5. Albuquerque, P.M.C. Abelhas silvestres (Hymenoptera, Apoidea) e suas fontes de alimento em um ecossistema de Dunas, na Ilha do Maranhão, MA, Brasil: composição, fenologia e interações. PhD Thesis, Universidade de São Paulo, Ribeirão Preto. (1998).
6. Andena, S.R., Bego, L.R., & Mechi, M.R. As comunidades de abelhas (Hymenoptera, Apoidea) de uma área de cerrado (Corumbataí, SP) e suas visitas às flores. *Revista Brasileira Zoociências*, **7**, 55-91. (2005).
7. Carvalho, C.A.L. Abelhas (Hymenoptera, Apoidea) no município Cruz das Almas - Bahia: levantamento, identificação e material coletado em plantas de importância econômica. Msc Thesis. Universidade Federal da Bahia. Salvador. (1993).
8. Carvalho, C.A.L. Diversidade de abelhas (Hymenoptera, Apoidea) e plantas visitadas no município de Castro Alves – BA. PhD Thesis. Universidade de São Paulo, Piracicaba. (1999).
9. Carvalho, C. A. L., Marques, O. M., & Sampaio, H. S. V. Abelhas (Hymenoptera, Apoidea) em Cruz das Almas - Bahia: 1. Espécies coletadas em fruteiras. *Insecta*, **4**, 11-17. (1995).
10. D'Avila, M. Insetos visitantes florais em áreas de cerradão e cerrado sensu stricto no Estado de São Paulo. PhD Thesis. Universidade de São Paulo, Piracicaba. (2006).

11. Faria, G.M. A flora e a fauna apícola de um ecossistema de campo rupestre, Serra do Cipó – MG, Brasil: Composição, fenologia e suas interações. PhD Thesis. Universidade de São Paulo. (1994).
12. Faria-Mucci, G.M., Melo, M.A., & Campos, L.A.O. A fauna de abelhas (Hymenoptera, Apoidea) e plantas utilizadas como fonte de recursos florais, em um ecossistema de campos rupestres em Lavras Novas, Minas Gerais, Brasil. Apoidea Neotropica (Melo, G. R., Alves-dos-Santos, I.). UNESC, Criciúma. (2003).
13. Knoll, F.R.N. Abundância relativa, sazonalidade e preferências florais de Apidae em uma área urbana (23º33'D; 46º43'W). PhD Thesis. Universidade de São Paulo, São Paulo. (1990).
14. Lima, M.F.C. Comunidade de abelhas, nidificação de abelhas solitárias em cavidades preexistentes (Hymenoptera: Apoidea) e interação abelha-planta na Reserva Biológica Guaribas, Mamanguape, Paraíba, Brasil. PhD Thesis. Universidade Federal da Paraíba, João Pessoa. (2004).
15. Mateus S. Abundância relativa, fenologia e visita às flores pelos Apoidea do cerrado da Estação Ecológica de Jataí - Luiz Antônio – SP. Msc Thesis. Universidade de São Paulo, Ribeirão Preto. (1998).
16. Pedro, S.R.M. Sobre as abelhas (Hymenoptera, Apoidea) em um ecossistema de cerrado (Cajuru, NE do Estado de São Paulo): composição, fenologia e visita às flores. Msc Thesis. Universidade de São Paulo, Ribeirão Preto. (1992).
17. Rêgo, M.M.C. Abelhas silvestres (Hymenoptera, Apoidea) em um ecossistema de cerrado s.l. (Chapadinha – MA, Brasil): uma abordagem biocenótica. PhD Thesis. Universidade de São Paulo, Ribeirão Preto. (1998).
18. Silva, M.C.M. Estrutura da comunidade de abelhas (Hymenoptera, Apoidea) de uma área de restinga (Praia de Intermares, Cabedelo, Paraíba, Nordeste do Brasil). Msc Thesis. Universidade Federal da Paraíba, João Pessoa. (1998).

19. Silva, M.C.M., & Martins, C.F. Flora apícola e relações tróficas de abelhas (Hymenoptera, Apoidea) em uma área de restinga (Praia de Intermares, Cabedelo - PB, Brasil). *Principia*, 7, 40-51. (1999).
20. Silveira, M.S. Fauna de abelhas (Hymenoptera, Apoidea, Apiformes) e recursos florais utilizados em áreas urbanas e no entorno da cidade de João Pessoa, PB. Msc Thesis. Universidade Federal da Paraíba, João Pessoa. (2006).
21. Sofia, S.H. As abelhas e suas visitas às flores em duas áreas urbanas. PhD Thesis. Universidade Estadual Paulista, Rio Claro. (1996).
22. Wilms, W. Die Bienenfauna im Küstenregenwald Braziliens und ihre Beziehungen zu Blütenpflanzen: Fallstudie Boracéia, São Paulo. PhD Thesis. Universität Tübingen, Tübingen. (1995).
23. Wilms, W., Imperatriz-Fonseca, V.L., & Engels, W. Resource partitioning between highly eusocial bees and possible impact of the introduced Africanized honey bee on native stingless bees in the Brazilian Atlantic Rainforest. *Studies on Neotropical Fauna and Environment*, **31**, 137-151. (1996).
24. Gastauer, M., Caldeira, C.F., Trotter, I., Ramos, S.J. & Meira Neto, J.A.A. Optimizing community trees using the open tree of life increases the reliability of phylogenetic diversity and dispersion indices. *Ecological Informatics*, **46**, 192–198. (2018)
25. Hinchliff, C.E., Smith, S.A., Allman, J.F., Burleigh, J.G., Chaudhary, R., Coghill, L.M., Crandall, K.A., *et al.* Synthesis of phylogeny and taxonomy into a comprehensive tree of life. *PNAS*, **112**, 12764 -12769. (2015)
26. Gastauer, M. & Meira-Neto, J.A.A. Updated angiosperm family tree for analyzing phylogenetic diversity and community structure. *Acta Bot. Bras.*, **31**, 191-198. (2017).
27. Revell, L.J. phytools: An R package for phylogenetic comparative biology (and other things). *Methods in Ecology and Evolution*, **3**, 217-223. (2012).

28. Webb, C.O. & Donoghue, M.J. (2005). Phylomatic: Tree assembly for applied phylogenetics. *Mol. Ecol. Notes*, **5**, 181-183.

29. Magallón S., Gómez-Acevedo, S., Sánchez-Reyes, L.L., Hernández-Hernández, T. (2015). A metacalibrated time-tree documents the early rise of flowering plant phylogenetic diversity. *New Phytologist*, **207**, 437-453.
